# Hog1 MAP kinase modulates early riboflavin accumulation under low-pH and saline conditions in *Debaryomyces hansenii*

**DOI:** 10.3389/fmicb.2026.1746023

**Published:** 2026-02-20

**Authors:** Diana Villarreal-Huerta, Benjamín Mendoza-Téllez, Miguel Ángel Rosas-Paz, Norma Silvia Sánchez, Raziel Arturo Jiménez-Nava, Eliseo Cristiani-Urbina, Claudia Segal-Kischinevzky, James González

**Affiliations:** 1 Departamento de Biología Celular, Facultad de Ciencias, Mexico City, Mexico; 2 Posgrado en Ciencias Biológicas, Mexico City, Mexico; 3 Posgrado en Ciencias Bioquímicas, Mexico City, Mexico; 4 Departamento de Genética Molecular, Instituto de Fisiología Celular, Universidad Nacional Autónoma de México, Mexico City, Mexico; 5 Departamento de Ingeniería Bioquímica, Escuela Nacional de Ciencias Biológicas, Instituto Politécnico Nacional, Unidad Profesional Adolfo López Mateos, Mexico City, Mexico; 6 Departamento de Microbiología, Escuela Nacional de Ciencias Biológicas, Instituto Politécnico Nacional, Mexico City, Mexico

**Keywords:** *Debaryomyces hansenii*, *DhHog1*, flavinogenic yeasts, riboflavin metabolism, HOG pathway, stress adaptation

## Abstract

Riboflavin (vitamin B2) is an essential precursor of flavin cofactors involved in redox metabolism, and its industrial production increasingly relies on microbial fermentation. *Debaryomyces hansenii* is a halotolerant flavinogenic yeast previously exploited for riboflavin biosynthesis; however, its biotechnological application has been limited by genetic instability and incomplete understanding of its regulatory networks. Here, we reveal a novel connection between the High Osmolarity Glycerol (HOG) pathway and riboflavin metabolism in *D. hansenii*. Using a stable *Dhhog1*Δ mutant, we demonstrate that loss of *Dh*Hog1 leads to earlier secretion of riboflavin under acidic and saline conditions, visible as a yellow fluorescent pigment in the culture medium. This early riboflavin accumulation was accompanied by altered assimilation of phosphorus, sulfur, and magnesium but not iron, suggesting that regulation extends beyond classical iron limitation. Gene expression analyses showed up-regulation of *RIB1*, *RIB4*, and *RIB6*, together with derepression of *SEF1*, indicating that *Dh*Hog1 modulates the timing of riboflavin production. These findings uncover a previously unrecognized role of the HOG pathway in coordinating stress responses with secondary metabolism and highlight *D. hansenii* as a promising platform for metabolic engineering of riboflavin production.

## 1 Introduction

*Debaryomyces hansenii* (previously as syn. *Candida famata*) is an ascomycetous yeast of the subphylum Saccharomycotina, belonging to the CTG (Ser1) clade, a group of yeasts that predominantly translate the CTG codon as serine instead of leucine (Prista et al., 2016; Krassowski et al., 2018; Ochoa-Gutiérrez et al., 2022). One of its most studied traits is halotolerance, as it can grow in media containing up to 4 M NaCl (Onishi, 1963), and is considered an extremotolerant yeast (Segal-Kischinevzky et al., 2022; González et al., 2025). This ability is largely mediated by the High Osmolarity Glycerol (HOG) pathway, a phosphorylation cascade that activates Hog1 MAP kinase (Bansal and Mondal, 2000; Sánchez et al., 2020). Phosphorylated Hog1 orchestrates adaptive responses, promoting glycerol accumulation and regulating osmoprotective genes (Posas et al., 1996; Hohmann, 2002, 2009; Sánchez et al., 2020; de la Fuente-Colmenares et al., 2024).

Compared with its close relative, *Candida albicans*, the production of stable mutants in *D. hansenii* has been hindered by a low occurrence of homology-directed repair of its DNA, as well as the scarcity of optimized genetic tools geared toward the modification of this specific yeast, due in part to the ambiguous translation of the CUG codon characteristic of the yeasts belonging to the CTG clade (Richard et al., 2005; Gomes et al., 2007; Minhas et al., 2009; Ochoa-Gutiérrez et al., 2022). The first stable null mutant, *Dhhog1*Δ, was developed for the further study of *D. hansenii*’s HOG pathway (Sánchez et al., 2020).

In addition to halotolerance, *D. hansenii* is a flavinogenic yeast, capable of synthesizing and secreting riboflavin (vitamin B2) (Gadd and Edwards, 1986). Riboflavin is a precursor of the flavin adenine dinucleotide (FAD) and flavin mononucleotide (FMN), which are essential for multiple physiological processes, including redox homeostasis, protein folding, DNA repair, fatty acid β-oxidation, amino acid oxidation, and choline metabolism (Bray et al., 1964; Frerman, 1988; Baron and Hylemon, 1995; Mansoorabadi et al., 2007; Macheroux et al., 2011; Walsh and Wencewicz, 2013). Unlike plants, fungi, and most prokaryotes, animals cannot synthesize riboflavin and must obtain it from their diet or, to a lesser extent, gut microbiota (LeBlanc et al., 2013; Nysten and Van Dijck, 2023). Therefore, industrial-scale production of riboflavin is of great importance. Biological synthesis has replaced chemical synthesis due to higher efficiency, reduced waste, lower energy requirements, and use of renewable substrates such as sugars or vegetable oils (Vandamme, 1992; Stahmann et al., 2000; Lim et al., 2001; Abbas and Sibirny, 2011; Schwechheimer et al., 2016). The global riboflavin market is projected to be valued at USD 508.6 million in 2025, with steady growth expected to reach USD 872.24 million by 2033, representing a compound annual growth rate (CAGR) of 6.98% during this period (Sharma, 2025).

The first report of *D. hansenii*’s flavinogenic capacity was made by Gadd and Edwards (Gadd and Edwards, 1986), when they noticed a yellow pigment in the supernatant of iron-depleted media. Riboflavin (vitamin B2) is a yellow, water-soluble compound whose green fluorescence can be detected within 440/535 nm excitation/emission wavelengths. Since then, iron limitation has become one of the most commonly used conditions in flavinogenic media, among others such as exposure to copper, cobalt, zinc, chromium, and industrial protein-rich wastewaters (Fedorovich et al., 1999; Fedorovych et al., 2001; Seda-Miró et al., 2007).

**FIGURE 1:**
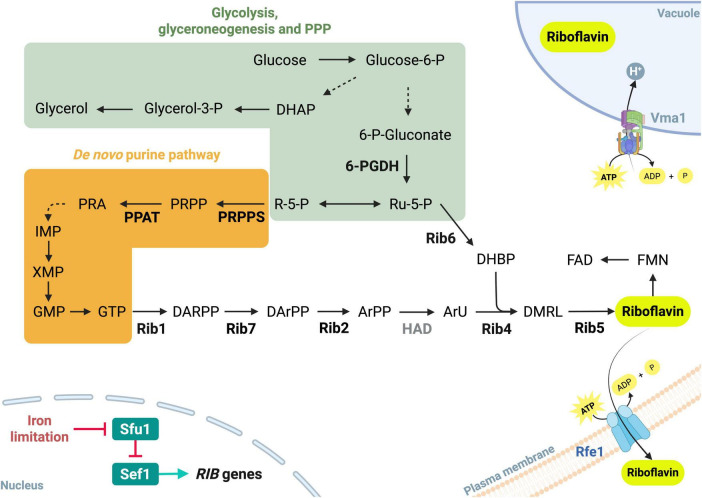
Simplified schematic of the riboflavin biosynthetic pathway in flavinogenic yeasts under iron limitation. The pathway is fueled by glycolysis and pentose phosphate pathway precursors (green square: 6-PGDH, 6-phosphogluconate dehydrogenase; R-5-P, ribose-5-phosphate; Ru-5-P, ribulose-5-phosphate). *PRS3* encodes 5-phosphoribosyl-1-pyrophosphate synthetase (PRPPS), responsible for PRPP (5-phosphoribosyl-1- pyrophosphate) production. The *ADE4* gene encodes 5-phosphoribosyl-1-pyrophosphate amidotransferase (PPAT), a key enzyme in purine biosynthesis that catalyzes the first step of the de *novo* pathway. Purine-derived precursors (orange square: PRPP, 5-phosphoribosyl-1- pyrophosphate; PRD, 5-phosphoribosylamine; IMP, inosine monophosphate; XMP, xanthosine monophosphate; GMP, guanosine monophosphate; GTP, guanosine triphosphate). Key genes/enzymes of riboflavin pathway include *RIB1*/Rib1 (GTP cyclohydrolase II), *RIB2/*Rib2 (DArPP deaminase), a putative haloacid dehalogenase/phosphatase (HAD), *RIB4/*Rib4 (dimethylribityllumazine synthase), *RIB5/*Rib5 (riboflavin synthase), *RIB6/*Rib6 (4-dihydroxy-2-butanone-4-phosphate synthase), and *RIB7/*Rib7 (5-amino-6-(5-phosphoribosylamino) uracil reductase). The intermediates in the riboflavin pathway include DARPP (2,5-diamino-6-ribosylamino-4(3H)-pyrimidinone 5′-phosphate), DArPP (5-amino-6-(D-ribitylamino) uracil 5′-phosphate), ArPP (5-amino-6-ribityl-amino-2,4(1H, 3H) pyrimidinedione 5′-phosphate), ArU (5-amino-6-(D-ribitylamino) uracil), DHBP (3,4-dihydroxy-2-butanone-4-phosphate), DMRL (6,7-dimethyl-8-ribityllumazine), FMN (flavin mononucleotide), and FAD (flavin adenine dinucleotide). In the vacuole, riboflavin can be stored through a process dependent on Vma1, the vacuolar ATPase subunit A. Riboflavin can also be secreted via Rfe1, a riboflavin excretase. Under iron homeostasis conditions, the GATA-type transcription factor Sfu1 prevents iron toxicity in iron-replete medium by inhibiting Sef1, a transcription factor that activates iron acquisition genes, including *RIB* genes. Blue arrows indicate positive regulation; red blunt arrows indicate negative regulation. Image based on findings reported in the literature (Voronovsky et al., 2002; Dmytruk et al., 2004; Voronovsky et al., 2004; Haase et al., 2013; Sarge et al., 2015; Sa et al., 2016; Ruchala et al., 2022, 2025). Created using BioRender.com, accessed on 29 August 2025.

The biosynthesis pathway of riboflavin has been elucidated in the *D. hansenii* synonym species *C. famata*. The pathway, which includes six *RIB* genes, involves sequential dephosphorylation and reduction reactions that convert GTP and ribulose-5-phosphate into riboflavin through several intermediates (Figure 1). Key enzymes include GTP cyclohydrolase II (Rib1), DArPP deaminase (Rib2), 6,7-dimethyl-8-ribityllumazine synthase (Rib4), riboflavin synthase (Rib5), 4-dihydroxy-2-butanone-4-phosphate synthase (Rib6), and 5-amino-6-(5-phosphoribosylamino) uracil reductase (Rib7) (Bacher et al., 2001; Voronovsky et al., 2004). In *C. famata*, the overexpression of *RIB1* and *RIB6* has proven effective for the overproduction of riboflavin (Petrovska et al., 2022).

Unlike *S. cerevisiae*, most yeasts belonging to the CTG clade possess a negative feedback loop involved in both iron and riboflavin metabolism. Sef1 is a transcriptional activator of iron acquisition and riboflavin biosynthesis genes that remains repressed by Sfu1 in iron-replete media (Chen et al., 2011; Andreieva et al., 2020a,b; Demuyser et al., 2020).

Iron limitation has been framed as a necessary condition to induce the excretion of riboflavin in most yeasts (Ruchala et al., 2025). In *Ashbya gossypii*, the induction of flavinogenesis has been linked to oxidative stress, UV-light exposure, sporulation, and endoplasmic reticulum stress (Stahmann et al., 2001; Nieland and Stahmann, 2013; Silva et al., 2018; Kurabayashi et al., 2025; Ruchala et al., 2025). In other yeasts, including *Candida albicans*, *Candida glabrata*, *Cryptococcus neoformans*, and *Saccharomyces cerevisiae*, Hog1 has been identified as a negative regulator of iron assimilation (Kaba et al., 2013; Lee et al., 2014; Srivastava et al., 2015; Martins et al., 2018). However, a direct connection between Hog1 signaling and riboflavin biosynthesis has not yet been established.

This study aims to elucidate the role of *Dh*Hog1 in riboflavin metabolism under saline condition, integrating genetic, biochemical, and physiological approaches to uncover the interplay between osmotic stress signaling and flavinogenesis in *D. hansenii*.

## 2 Materials and methods

### 2.1 Strains and growth conditions

Precultures of *D. hansenii* wild-type strain CBS767 (WT) and the isogenic mutant *Dhhog1*Δ, generated by Sánchez et al. (2020) were grown and maintained on Yeast Peptone Dextrose agar (YPD agar; 1% yeast extract, 2% peptone, 2% glucose, and 2% agar). Liquid precultures were prepared in YPD medium using a flask-to-medium ratio of 2:5, incubated at 28°C with shaking at 180 rpm overnight, representing the seed condition (time 0 h). Cells were recovered by centrifugation at 3,000 rpm for 5 min, washed twice, and resuspended in sterile distilled water. Subsequently, cultures were grown in minimum media based on Yeast Nitrogen Base (YNB) supplemented with 2% glucose, 0.5% ammonium sulfate as the sole nitrogen source, and 0.6 M NaCl (from now on referred to as minimum media + 0.6 M NaCl).

### 2.2 Growth curves and pH measurements

Washed preculture cells were used as an inoculum for the minimum medium at an initial optical density of 0.05 (measured at 600 nm, OD_600__*nm*_). Successive OD_600__*nm*_ measurements were taken using a Beckman Coulter DU^®^ 640 spectrophotometer. For the flavinogenic condition (minimum media + 0.6 M NaCl, initial pH 4.3), the exponential growth phase was defined at OD_600__*nm*_ = 0.5 (18–20 h), whereas the stationary growth phase was defined at OD_600__*nm*_ = 3.8–4.0 (48 h).

For pH experiments, the basal pH of the minimum media YNB (initially 4.3) was adjusted to 6.8–7.0 using 2 N NaOH. pH measurements were carried out at defined time intervals with a potentiometer (pH 210, Microprocessor pH Meter, Hanna Instruments), previously calibrated according to the manufacturer’s instructions.

### 2.3 Riboflavin measurements

Extracellular riboflavin was quantified by measuring fluorescence intensity at emission/excitation wavelengths of 440/535 nm. A riboflavin stock solution (20.1 mg/L) was prepared by dissolving riboflavin powder in distilled water under mild heating (<30°C). Culture aliquots (15 mL) were collected at multiple time points ranging from 24 to 122 h to capture the onset and dynamics of riboflavin production, as indicated by the fluorescence and yellow coloration of the supernatant, covering both exponential and stationary growth phases and the associated pH decrease. Aliquots were centrifuged, and supernatant was filtered through 0.22 μm pore-size filters. Subsequently, 200 μL of the filtered supernatants were transferred to black 96-well Costar^®^ assay plates with clear flat bottoms, and fluorescence was measured with a Synergy H1 Microplate Reader (Biotek), gain = 50. Riboflavin concentrations were determined by interpolation against a standard curve generated from 1:2 serial dilutions of an initial 10.05 mg/L riboflavin solution.

### 2.4 Riboflavin extraction and RP-HPLC-DAD analysis

Sterile culture supernatants were lyophilized to concentrate the fluorescent compound and remove water. The resulting dried powder was extracted with 50 mL HPLC-grade methanol and vortexed for 5 min at room temperature (25 ± 1.0°C). Extracts were centrifuged at 3,500 rpm for 10 min, and the methanolic phase was recovered and dried at 35°C for 72 h. Reversed-phase high-performance liquid chromatography (RP-HPLC) coupled with a Diode Array Detector (DAD), was then carried out, following the method described by Jiménez-Nava et al. (2023). Methanolic residues were resuspended in 10 mL HPLC-grade water, and 2 mL aliquots were loaded in triplicate onto C18 solid-phase extraction (SPE) cartridges (Alltech^®^ Maxi-Clean™, Thermo Fisher Scientific, Waltham, MA, United States). Cartridges were eluted under vacuum with 1.2 mL of HCl-acidified methanol (0.002% v/v). Eluates were analyzed using an Agilent 1,260 Infinity Series system equipped with a DAD, monitoring 280 and 440 nm, with a Zorbax SB-Phenyl column (150 × 4.6 mm, 5 μm, Agilent Technologies, Santa Clara, CA, United States). The mobile phase was acetonitrile and 0.05% phosphoric acid in water at 0.5 mL/min, applying a linear gradient of 10–100% acetonitrile over 15 min.

Absorption spectra of the HPLC-separated fractions were obtained directly from DAD data and visualized with Agilent ChemStation software, using a riboflavin standard (Sigma-Aldrich, Cat. No. 47861) for reference.

### 2.5 Element analysis

Culture supernatants (15 mL) were collected at 18 h and 48 h from independent biological cultures and clarified at room temperature by centrifugation (3,000 rpm, 5 min). Clarified supernatants were transferred to clean tubes and filtered through 0.22 μm membranes prior to analysis. No acid digestion was performed; therefore, measurements correspond to the dissolved fraction of the clarified supernatant. Elemental quantification was performed by Inductively Coupled Plasma–Optical Emission Spectroscopy (ICP-OES) by an external analytical service using the proprietary Triton ICP-OES Test (TRITON Applied Reef Bioscience, Germany).^1^ Due to the proprietary nature of the service, detailed instrument parameters are not disclosed by the provider, and results are reported as a single quantitative concentration output per submitted sample. Elements quantified included B, Ba, Be, Br, Ca, Cl, Co, Cr, Cu, F, Fe, I, K, Li, Mg, Mn, Mo, Na, Ni, P, PO4, S, Si, Sr, V, and Zn (Supplementary Table 1). Element concentrations are reported in mg/L or μg/L and converted to μM (Supplementary Table 1). Detection limits were those provided by the facility, and all reported values were above the sensitivity threshold.^2^ All samples were analyzed under identical matrix conditions (same culture medium, pH, and NaCl concentration), enabling direct comparisons between strains and time points.

### 2.6 RNA extraction

Total RNA was extracted following the protocol described by Schmitt et al. (1990). Cultures were first grown overnight in rich medium (YPD) to obtain seed cultures (time 0 h), and subsequently inoculated into minimum media with 0.6 M NaCl. Cells were harvested at exponential phase and stationary phase.

Briefly, cells were washed twice, centrifuged at 3,000 rpm, and mechanically disrupted using a vortex mixer with sterile glass microbeads (425–600 μm) previously incubated in phenol (pH 4.5). Samples were incubated at 65°C for 5 min and vortexed twice for 30 s. The mixture was chilled and centrifuged to separate the aqueous and phenolic phases. The aqueous phase was extracted twice with phenol:chloroform:isoamyl alcohol (25:24:1) and chloroform:isoamyl alcohol (24:1). RNA was precipitated by adding 1/10 volumes of sodium acetate (3 M, pH 5.2) and 2.5 volumes of absolute ethanol, followed by incubation at −20°C for 30 min and centrifugation at 14,000 rpm. The resulting pellet was washed, dried, and resuspended in RNase-free water. RNA integrity was assessed by electrophoresis on a 1% denaturing agarose gel.

### 2.7 Identification of riboflavin biosynthesis genes

The *D. hansenii* genome remains only partially annotated, with most gene assignments based on *in silico* predictions of orthologous genes. To validate these annotations, multiple sequence alignments of selected riboflavin biosynthesis proteins and the transcription factor Sef1 were carried out against their orthologous in *Saccharomyces cerevisiae* and *Candida albicans* (Supplementary Material 1). Amino acid sequences were further compared to confirm that the predicted ORFs corresponded to the expected protein functions (*RIB1*, locus DEHA2A12870g; *RIB2*, locus DEHA2E11374g; *RIB4*, locus DEHA2D04180g; *RIB5*, locus DEHA2D13926g; *RIB6*, locus DEHA2G09504g; *RIB7*, locus DEHA2G10010g; *SEF1*, locus DEHA2C16676g). Protein identifiers (IDs) were retrieved from the NCBI Protein Database. Both analyses were performed within the NCBI BlastP Tool.

### 2.8 *In silico* predictions of transcription factor binding sites

The intergenic region spanning from −625 bp upstream to the start codon of each gene was scanned with position-specific scoring matrices (PSSMs) using the Regulatory Sequence Analysis Tools (RSAT) platform,^3^ employing the full matrix-scan tool within the Fungi Server (Turatsinze et al., 2008). Binding motifs for Hog1-regulated transcription factors were identified, including Sef1 (ID: 2900038, locus DEHA2C16676g), Hot1 (ID: 2901482, locus DEHA2D15136g), Sko1 (ID: 2901375, locus DEHA2D09196g), Skn7 (ID: 2913769, locus DEHA2B08052g), Msn2/4 (ID: 2899670, locus DEHA2A08382g), and Yap1 (ID: 2904514, locus DEHA2G02420g).

Except for Sef1 and Hot1, motif matrices were retrieved from the JASPAR database, specifically from the 2024 core fungi collection: Sko1 (ID: MA0382.1), Skn7 (ID: MA0381.1), Msn2/4 (ID: MA0341.1/MA0342.1), and Yap1 (ID: MA0415.1). A *D. hansenii*-specific background model estimation method with a Markov order of 1 was applied, and a significance threshold of *p*-values < 0.0006 was used for significant determination. Putative Sef1 binding sites were searched manually in each intergenic region based on the DNA recognition motifs identified by Chen et al. (2011) and Romanov et al. (2025) for *C. albicans* and *C. famata* Sef1, respectively. Two distinct putative Hot1 binding sites were searched manually based on the motifs reported by Cook and O’Shea (2012), Bai et al. (2015) and Gomar-Alba et al. (2015) for *S. cerevisiae* (Supplementary Figure 2). The Sef1 consensus binding motif logo was derived from multiple alignments of the upstream intergenic regions of *RIB* genes with reported sequences from *C. albicans* and *C. famata* (Chen et al., 2011; Romanov et al., 2025; Supplementary Figures 3, 4).

### 2.9 Analysis of gene expression

To preserve RNA integrity and minimize RNase contamination during downstream gene expression analyses, all RNA handling steps were performed under RNase-free conditions using RNase-free plasticware and reagents. RNA integrity was assessed by agarose gel electrophoresis prior to reverse transcription. For RT-qPCR analyses, total RNA samples were treated with DNase I to remove residual genomic DNA before cDNA synthesis. First-strand cDNA was synthesized using the Thermo Scientific RevertAid H Minus Kit (K1632), which includes the RiboLock RNase inhibitor to protect RNA during reverse transcription. The resulting cDNA was subsequently used as a template for quantitative PCR (qPCR).

RT-qPCR assay was performed using the standard curve method with gene-specific deoxyoligonucleotides designed for the genes encoding Hog1 MAPK (*DhHOG1*), sugar transporter-like protein (*DhSTL1*), the transcriptional activator Sef1 (*DhSEF1*), GTP cyclohydrolase II (*DhRIB1*), DArPP deaminase (*DhRIB2*), 6,7-dimethyl-8-ribityllumazine synthase (*DhRIB4*), riboflavin synthase (*DhRIB5*), 4-dihydroxy-2-butanone-4-phosphate synthase (*DhRIB6*), 5-amino-6-(5-phosphoribosyl-amino) uracil reductase (*DhRIB7*). The actin gene (*DhACT1*) was used as the housekeeping control.

Deoxyoligonucleotides were evaluated to ensure the absence of dimer formation and cross-hybridization, and only deoxyoligonucleotides pairs with amplification efficiencies > 90% were used (Table 1). RT-qPCR was performed with a Rotor-Gene Q thermocycler (Qiagen) using SYBR Green as the detection dye (KAPA SYBR Fast Kit, Roche). Cycling conditions were: 94°C for 5 min (1 cycle), followed by 35 cycles of 94°C for 15 s, 60°C for 20 s, and 72°C for 20 s.

**TABLE 1:** Deoxyoligonucleotides used for RT-qPCR relative gene expression analysis.

Gene	ID, ORF	Fw 5′→ 3′	Rv 5′→ 3′
*DhACT1*	2901278, DEHA2D05412 g	CCCAGAAGAACACCCAGTTT	CGGCTTGGATAGAAACGTAGAA
*DhRIB1*	2899385, DEHA2A12870 g	AAGACACCCTGCTGATGGTC	TGTCGGGGTTGTTGGTCAAT
*DhRIB2*	2902834, DEHA2E11374 g	TGGAACCATGCTCCTTGAGATT	CTGGCTCCACAACACCAACA
*DhRIB4*	2901083, DEHA2D04180 g	TGTTTGACCGATGAGCAAGC	ACACATTTCGACAGCAGCAG
*DhRIB5*	2901307, DEHA2D13926 g	GCCTGGGTGTAACTGACCAT	GGAGAAGGGGTTCATTGCCA
*DhRIB6*	2904849, DEHA2G09504 g	TGGTCTTATGAAGTCTACCGGC	TATGCTGATGGCACGACCAC
*DhRIB7*	2904875, DEHA2G10010 g	ACTTGCACCTCCTTCAACCAT	GGTGCATTTGTCAGGCTTCC
*DhSEF1*	2900038, DEHA2C16676 g	CCGTTTGCTTCGACCCTTTA	CTGCCAACAATGCTACCGTG
*DhSTL1*	2902951, DEHA2E01364 g	TGGGAATGGCTGACACTTATG	GCTCTTCTACCCAACCTATCAATC
*DhHOG1*	2902985, DEHA2E20944 g	AACCGCTCGCTGAATGGAAT	TCTCCACCTCCAGACGTGAT

Relative transcript levels were normalized to the WT mean Ct for each gene using the 2^∧−^ΔΔCt method, and data were expressed as fold change values. Results represent mean ± SD from three biological replicates, each analyzed in duplicate (technical replicates).

### 2.10 Statistical analysis

Unpaired *t*-tests were applied to compare the means of the WT and *Dhhog1*Δ groups within each time point or gene in GraphPad Prism (GraphPad Software Inc.). Optical density, pH, riboflavin quantification and relative expression means were based on three independent experiments (*n* = 3). For riboflavin quantification and gene expression, statistical significance was indicated as follows: *p* ≤ 0.05 (*), *p* ≤ 0.005 (**), *p* ≤ 0.0005 (***), and *p* ≤ 0.00005 (****). Results are presented as the mean of three independent measurements ± standard deviation (SD).

For *in silico* predictions of transcription factor binding sites (TFBS), a *D. hansenii*-specific background model estimation method with a Markov order of 1 was applied, and a significance threshold of *p*-values < 0.0006 was used for the determination of putative binding sites in select riboflavin-related genes.

## 3 Results

### 3.1 Initial low pH induces the excretion and accumulation of riboflavin in *D. hansenii*

**FIGURE 2:**
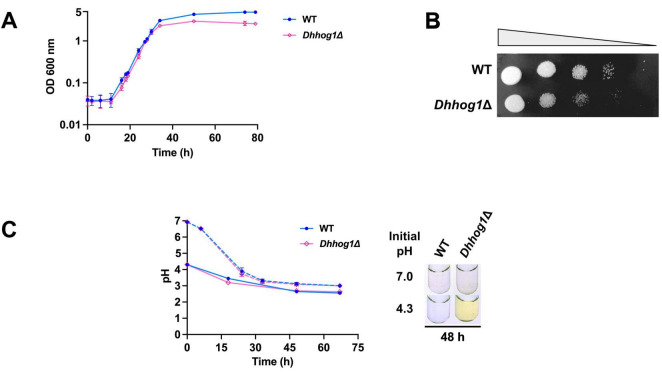
Initial low pH induces the secretion and accumulation of a yellow pigment in the culture supernatant of *D. hansenii*. **(A)** Growth curves of wild type (WT, blue) and the *Dhhog1*Δ mutant (pink) were determined in minimum media + 0.6 M NaCl at an initial low pH (pH 4.3). **(B)** Cells of WT and the *Dhhog1*Δ mutant were grown in minimum media + 0.6 M NaCl to the stationary phase (74 h), followed by 10-fold serial dilutions (up to 10^–4^). A 10μL aliquot of each dilution was spotted onto YPD agar plates containing 0.6 M NaCl and incubated at 28°C for 3 days. **(C)** The pH of cultures grown in minimum media supplemented with 0.6 M NaCl, initiated under neutral (pH 6.8–7.0, dotted lines) and acidic (pH 4.3, solid lines) conditions, was monitored over a 70 h period. Representative images from three independent experiments are shown. Data represent the mean ± standard deviation (SD) of three independent experiments (*n* = 3).

The high-osmolarity glycerol (HOG) pathway, with the MAP kinase Hog1 as its terminal effector under salt stress, is recognized as the most conserved signaling cascade for stress adaptation in fungi (de Nadal et al., 2002; Hohmann, 2002; Hohmann et al., 2007; Hohmann, 2009; Saito and Posas, 2012). Given previous observations of riboflavin efflux in acidic media (Perl et al., 1976; Vanetti and Aquarone, 1992), we investigated whether the initial medium pH modulates growth and riboflavin excretion in *D. hansenii* under saline stress, raising the possibility that *Dh*Hog1 signaling may also influence riboflavin metabolism. Comparative assays were conducted between the WT strain and the *Dhhog1*Δ mutant in minimum media (YNB) with glucose (2%) supplemented with 0.6 M NaCl under either neutral or acidic initial pH conditions. Significant differences in biomass and cell viability were observed during the stationary phase (Figures 2A,B), highlighting the role of *Dh*Hog1 in maintaining cellular fitness under osmotic stress.

**FIGURE 3:**
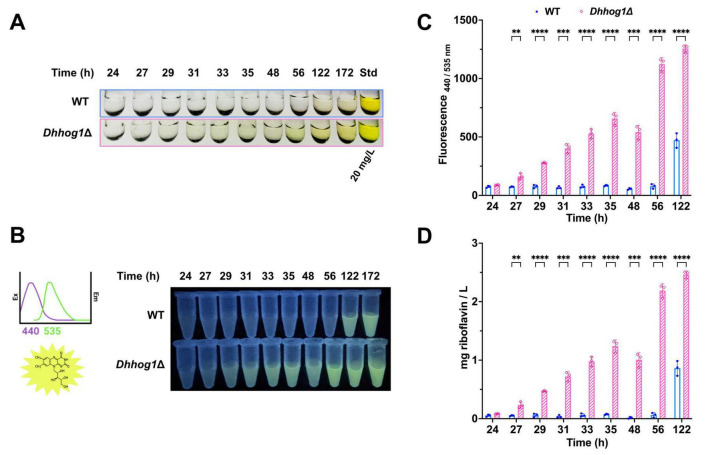
Quantification of riboflavin in the culture supernatant of *D. hansenii*. **(A)** A yellow pigment was observed in the supernatants from the WT and *Dhhog1*Δ cultures; the color appeared earlier in the culture time in the *Dhhog1*Δ strain. **(B)** Samples were collected at defined time points (24–172 h) from WT and *Dhhog1*Δ strains grown in minimum media + 0.6 M NaCl at an initial low pH (4.3) and excited with a UV light transilluminator. **(C,D)** Quantification was performed by measuring fluorescence (440/535 nm) and comparing with a calibration curve generated from a riboflavin standard in a 96-well spectrofluorometer. Data represent the mean ± standard deviation (SD) of three independent experiments (*n* = 3). Significant differences: *p* ≤ 0.05 (*), ≤ 0.005 (**), ≤ 0.0005 (***), ≤ 0.00005 (****).

Remarkably, cultures initiated at low pH exhibited secretion of a yellow pigment as early as the stationary phase in the *Dhhog1*Δ mutant (48 h) (Figure 2C), whereas in the WT strain, pigment accumulation became evident only at the late stationary phase (122–172 h) (Figure 3A). By contrast, no pigment secretion was detected in either strain when cultures were initiated at neutral pH (Figure 2C), even after 5 days of growth. Thus, NaCl was not sufficient to trigger pigment secretion at neutral pH, supporting a cooperative effect between acidic pH and salinity. These findings indicate that initial acidic conditions are a key determinant for pigment production and excretion in *D. hansenii*, with an accelerated onset in the absence of *HOG1*.

**FIGURE 4:**
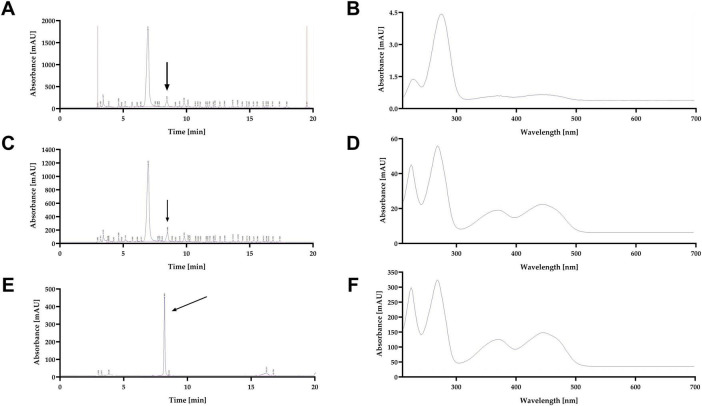
RP-HPLC-DAD analysis and UV-Vis spectra of riboflavin in *D. hansenii* WT and *Dhhog1*Δ. Chromatograms at 280 nm obtained by RP-HPLC-DAD for WT **(A)**, *Dhhog1*Δ **(C)**, and riboflavin standard **(E)**, with arrows indicating the HPLC-separated fraction whose UV-Vis spectra are shown in **(B,D,F)**. Samples were collected during stationary phase from strains grown in minimum media + 0.6 M NaCl at initial pH 4.3. Riboflavin accumulation was higher in the *Dhhog1*Δ mutant compared with WT. Values represent the mean of at least three independent measurements.

Previous studies have reported that *D. hansenii* is capable of producing riboflavin, a metabolite responsible for the characteristic yellow coloration (Gadd and Edwards, 1986; Seda-Miró et al., 2007). Based on this evidence, we hypothesized that the yellow pigment observed under our culture conditions could correspond to riboflavin. To test this, we identify the pigment by RP-HPLC-DAD analyses in the culture supernatants from the *Dhhog1*Δ mutant, revealing the presence of a major fluorescent compound. Retention times closely matched those of a riboflavin standard (8.450 ± 0.003 min). The *Dhhog1*Δ mutant exhibited clearer chromatographic separation and UV-Vis absorption peaks characteristic of flavins (λmax = 223, 268, 370, 445 nm), consistent with riboflavin identity. In contrast, WT supernatants displayed weaker signals with partially overlapping absorption peaks. Representative chromatograms and UV-Vis spectra are shown in Figures 4A–F.

Furthermore, we examined the fluorescence properties of the riboflavin by recording excitation and emission spectra at the characteristic wavelengths (excitation: 440 nm; emission: 520–535 nm). Extracellular riboflavin production was monitored over time (24–172 h) (Figure 3A) to capture its onset and dynamics, as indicated by fluorescence and the characteristic yellow coloration of the supernatant. Sampling covered the exponential and stationary growth phases. Riboflavin concentrations were quantified by fluorimetry using a standard calibration curve (Figures 3B–D). These experiments confirm that the accumulation of riboflavin in the supernatant of cultures initiated at low pH, accumulates more rapidly over time in the *Dhhog1*Δ mutant. This observation indicates, for the first time, that *Dh*Hog1 is involved in the timing of riboflavin accumulation in *D. hansenii* under acidic and saline conditions.

### 3.2 Accelerated uptake of essential elements in *Dhhog1*Δ cells

**FIGURE 5:**
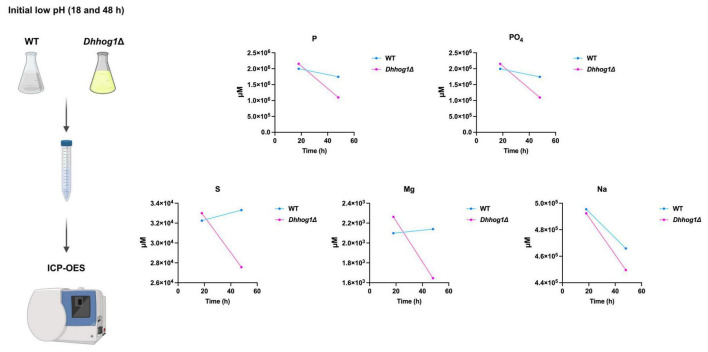
Identification and quantification of macro nutrients and trace elements in the culture supernatant of *D. hansenii* WT and *Dhhog1*Δ strains. Supernatants were collected from WT and *Dhhog1*Δ cultures grown in minimum media + 0.6 M NaCl at an initial low pH (4.3) at 18 and 48 h, and analyzed by Inductively Coupled Plasma-Optical Emission Spectroscopy (ICP-OES). A total of 43 elements (plus phosphate) were measured; the profiles of phosphorus (P), phosphates (PO_4_), sulfur (S), magnesium (Mg), and sodium (Na), are shown. Values (μM) were obtained from ICP-OES external service reports and are presented as quantitative profiles.

Iron limitation and the availability of other essential elements have been reported as key triggers for riboflavin excretion in riboflavin-producing yeasts (Averianova et al., 2020; Ruchala et al., 2025). To determine whether differences in elemental uptake contribute to the enhanced riboflavin accumulation observed in the *Dhhog1*Δ mutant, culture supernatants from 18 and 48 h under saline conditions and initial low pH were analyzed by Inductively Coupled Plasma Optical Emission Spectroscopy (ICP-OES) (Figure 5). Absolute concentrations (mg/L) and molar conversions (μM) are provided in Supplementary Table 1. Elemental profiles are interpreted as quantitative trends supporting comparisons between strains and time points.

By 48 h, the *Dhhog1*Δ mutant displayed more rapid assimilation of several essential elements, including phosphorus, phosphates, sulfur, and magnesium, compared to the WT strain. Notably, the most pronounced differences were observed for phosphorus/phosphate (1.75E+06/1.74E+06 μM), sulfur (3.22E+04 μM), and magnesium (2.14E+03 μM), consistent with accelerated assimilation of macronutrients in the *Dh*hog1Δ strain (1.10E+06/1.09E+06, 2.76E+04, and 1.65E+03 μM) during the transition to stationary phase (Figure 5). In contrast, uptake of iron and other elements was similar in both strains (Supplementary Table 1), indicating that mechanisms other than iron uptake are likely responsible for stimulating riboflavin excretion in *D. hansenii*. These results suggest that accelerated assimilation of specific elements (P, S, and Mg) in the *Dhhog1*Δ mutant may contribute to the earlier and higher riboflavin production observed under acidic and saline conditions.

### 3.3 Riboflavin biosynthesis genes are upregulated in the *Dhhog1*Δ mutant

The riboflavin biosynthesis genes *RIB1*, *RIB2*, *RIB5*, *RIB6*, and *RIB7* were previously cloned from the synonymous species *Candida famata* (Voronovsky et al., 2002, 2004; Dmytruk et al., 2004), and their sequences are available in NCBI GenBank as part of the Genolevures Consortium’s annotated reference genome for *D. hansenii* (ASM644v2). The *RIB4* gene sequence was inferred from its homologs in *S. cerevisiae* and *C. albicans* and incorporated into the Genolevures Consortium reference genome (Morgunova et al., 2007; Skrzypek et al., 2017). Amino acid sequence alignments confirmed the identity of the main riboflavin biosynthetic enzymes and revealed the degree of sequence conservation among them (Table 2).

**TABLE 2:** Sequence similarity and query coverage of selected *D. hansenii* riboflavin biosynthesis proteins compared with *S. cerevisiae* and *C. albicans*.

Protein name, Identifier	*S. cerevisiae*		*C. albicans*	
	Similarity (%)	Query cover (%)	Similarity (%)	Query cover (%)
*Dh*Rib1, DEHA2A12870p	77	80	85	92
*Dh*Rib2, DEHA2E11374p	73	86	80	100
*Dh*Rib4, DEHA2D04180p	83	99	95	100
*Dh*Rib5, DEHA2D13926p	73	98	82	100
*Dh*Rib6, DEHA2G09504p	73	98	88	100
*Dh*Rib7, DEHA2G10010p	62	100	65	100
*Dh*Sef1, DEHA2C16676p	67	76	70	100

**FIGURE 6:**
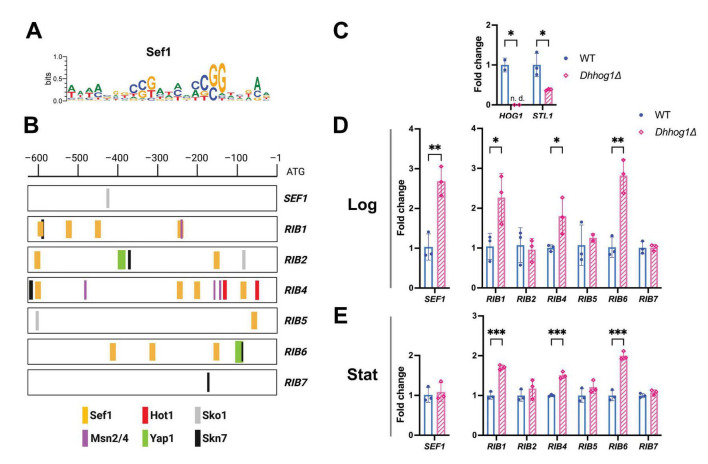
Predicted stress-related transcription factor binding sites in the promoter regions of riboflavin biosynthesis genes and corresponding gene expression in WT and *Dhhog1*Δ strains. **(A)** Sequence logo representing putative *SEF1* binding motifs derived from multiple alignments of the upstream intergenic regions of *RIB* genes with reported sequences from *Candida* spp. (Chen et al., 2011; Romanov et al., 2025). **(B)** Schematic representation of intergenic region from −625 bp upstream to the ATG of *SEF1/RIB* genes; predicted motifs are highlighted for Sef1 (yellow), Hot1 (red), Sko1 (gray), Msn2/4 (purple), Yap1 (green), and Skn7 (black). **(C)** As an initial control, *HOG1* and *STL1* expression were detected in WT (blue) and *Dhhog1*Δ (pink) strains. As expected, *HOG1* expression was not detected (n.d.) in the mutant, whereas partial *STL1* expression was observed. **(D,E)** Gene expression analysis of *SEF1*, *RIB1*, *RIB2*, *RIB4*, *RIB6*, and *RIB7* under minimum media + 0.6 M NaCl at an initial low pH in WT and *Dhhog1*Δ strains. Total RNA was extracted from WT and mutant cells during the mid-log phase (Log, 18 h) and stationary phase (Stat, 48 h) and analyzed by RT-qPCR. Bars represent fold changes in gene expression, with transcript levels normalized to *ACT1*. Values are presented as the mean of three independent measurements ± (SD). Significant differences: *p* ≤ 0.05 (*), ≤ 0.005 (**), ≤ 0.0005 (***).

To investigate the potential regulation of riboflavin biosynthesis genes by *Dh*Hog1 in *D. hansenii*, we performed an *in silico* analysis to identify putative stress-related transcription factor (TF) binding sites within the promoters of *RIB* genes and the transcription factor Sef1 (Figures 6A,B). Initially, a motif analysis was performed, and sequence logos were generated to represent putative *SEF1* binding motifs derived from multiple alignments of the upstream intergenic regions of *RIB* genes (Figure 6A) with previously reported sequences from *Candida albicans* and *C. famata* (Chen et al., 2011; Romanov et al., 2025). For the analysis of additional motifs, we relied on the previous work by de la Fuente-Colmenares et al. (2024), which validated the likely conservation of function of Sko1, Skn7, Msn2/4, and Yap1 proteins in *D. hansenii* through *in silico* comparisons with their homologs in *S. cerevisiae* and *C. albicans* (de la Fuente-Colmenares et al., 2024).

Our *in silico* intergenic region analysis revealed that the *RIB* gene promoters contain putative binding motifs for Hog1-controlled transcription factors Hot1, Sko1, Msn2/4, Yap1, and Skn7, as well as for the iron metabolism regulator Sef1 (Figure 6B). Specifically, Sef1-binding sequences were found predominantly in the promoters of *RIB1*, *RIB2*, *RIB4*, and *RIB6*. The two reported variants of Hot1-binding sequences in *S. cerevisiae* (Cook and O’Shea, 2012; Bai et al., 2015; Gomar-Alba et al., 2015) were only found in the *RIB4* promoter at a distance of 69 nt of each other. Sko1 motifs were present in *RIB2*, *RIB5*, and *SEF1* promoters. Skn7-binding sequences were detected in *RIB1*, *RIB2*, *RIB4*, and *RIB7*. Msn2/4 motifs were found in *RIB1* and *RIB4*, and Yap1-binding sites in *RIB2* and *RIB6*.

The absence of *HOG1* expression was confirmed in the *Dhhog1*Δ mutant, accompanied by reduced expression of *STL1*, a downstream *HOG* pathway gene involved in glycerol symport (Figure 6C). Expression profiles further reveal that, in the absence of *Dh*Hog1; *SEF1*, *RIB1*, *RIB4*, and *RIB6* transcripts accumulate at higher levels during the logarithmic phase, with upregulation of *RIB1*, *RIB4*, and *RIB6* genes persisting into the stationary phase in the *Dhhog1*Δ mutant (Figures 6D,E). Interestingly, *RIB2* and *RIB7* did not show increased expression despite riboflavin oversynthesis (Figures 6D,E). This suggests possible differential regulation of these genes, which may involve promoter-specific mechanisms or post-transcriptional control. These findings indicate that *Dh*Hog1 influences riboflavin biosynthesis in *D. hansenii*, possibly through the coordinated activity of stress-responsive transcription factors such as Hot1, Sko1, Skn7, Msn2/4, and/or Yap1, together with the iron metabolism regulator Sef1.

## 4 Discussion

The MAP kinase *Dh*Hog1 is well known for its central role in the high-osmolarity response in *Debaryomyces hansenii*, coordinating glycerol accumulation and catalase expression under saline and oxidative stress (Sánchez et al., 2020; de la Fuente-Colmenares et al., 2024). Our results reveal a link between *Dh*Hog1 and riboflavin metabolism. The *Dhhog1*Δ mutant displayed premature riboflavin secretion under acidic pH and NaCl, indicating that *Dh*Hog1 participates in the integration of pH and osmotic signals that normally delay riboflavin secretion until the stationary phase.

Riboflavin production is conserved in *D. hansenii* and closely related species, including *D. fabryi*, *D. subglobosus*, and *D. prosopidis* (Nguyen et al., 2009; Dmytruk et al., 2011; Dmytruk et al., 2014; Averianova et al., 2020; Ruchala et al., 2025). The higher levels of riboflavin observed in *Dhhog1*Δ are consistent with a *Dh*Hog1 influence on the expression of biosynthetic genes *RIB1*, *RIB4*, and *RIB6*, as well as the transcription factor *SEF1*. This observation suggests that similar regulatory mechanisms may be conserved and could operate in a comparable manner across other riboflavin-producing yeast species. Hog1 MAPK signaling is broadly conserved across yeasts; however, to our knowledge, this is the first report linking loss of *HOG1* to premature extracellular riboflavin accumulation in a riboflavinogenic yeast. In this regard, generating MAP Kinase pathway mutants, such as *hog1*Δ in other riboflavinogenic yeasts would be of particular interest to determine whether this regulatory mechanism is conserved, providing valuable insights into the evolutionary role of Hog1 in the control of riboflavin biosynthesis.

Although the reason behind the heterogeneous level of expression of the individual *RIB* genes remains elusive, the non-simultaneous induction of the complete set of *RIB* genes has been observed in earlier studies in *D. hansenii*, *C. famata* and *A. gossypii* (Ledesma-Amaro et al., 2015; Tsyrulnyk et al., 2020; Weintraub et al., 2025). Except for *RIB2*, our expression profile shares the upregulation of *RIB1*, *RIB4* and *RIB6* previously observed in *D. hansenii* under salt stress (Weintraub et al., 2025), as well as the marked expression of *AgRIB4* and, to a lesser extent, *AgRIB1* and *AgRIB3* (*RIB6* in *D. hansenii*) in *A. gossypii* compared to the other *AgRIB* genes in a WT strain (Ledesma-Amaro et al., 2015). Furthermore, single-gene overexpression and co-expression analysis done in *C. famata*, *A. gossypii*, and *Pichia pastoris* show a differential contribution of each of the *RIB* genes on riboflavin overproduction: in all the evaluated mutants, *RIB1* is the main bottleneck, and the simultaneous expression of *RIB1* and *RIB3/RIB6* is frequently observed in the overproducing strains (Marx et al., 2008; Ledesma-Amaro et al., 2015; Tsyrulnyk et al., 2020). The apparent discrepancy, where *RIB2* and *RIB7* did not show increased expression despite riboflavin oversynthesis in this study, could be explained by the known stoichiometry and regulation of the riboflavin pathway. Riboflavin biosynthesis requires two molecules of ribulose-5-phosphate (Ru5P) and one molecule of GTP (Chatwell et al., 2006), meaning that flux from the pentose phosphate pathway approximately doubles that of the purine pathway (Schlösser et al., 2001, 2007). In chemostatic cultures of *A. gossypii*, the transcript levels of *AgRIB6*, *AgRIB4*, and *AgRIB5* increase during phases of maximal riboflavin production, while *AgRIB2* and *AgRIB7* remain essentially constant, as they are generally constitutively expressed (Schlösser et al., 2007). Therefore, the upregulation of *RIB1*, *RIB4*, and *RIB6* in our study is consistent with the expected regulatory response to metabolic and stress conditions, whereas the lack of induction of *RIB2* and *RIB7* further supports their constitutive expression pattern.

Although the aim of the ICP-OES analysis was to address the iron contents in the supernatant, no differences were found between the WT and mutant strains; nevertheless, accelerated uptake of phosphorus, sulfur and magnesium was observed in the mutant. Determining the causes of this differential element uptake requires further investigation, but there is compelling evidence of the stimulating effect of Na^+^ cations and low pH on the respiratory activity of *D. hansenii* (Thomé-Ortiz et al., 1998; Almagro et al., 2000; Sánchez et al., 2008; Cabrera-Orefice et al., 2010; Guerrero-Castillo et al., 2011; Garcia-Neto et al., 2017; Navarrete et al., 2021, 2022), which could lead to an increased demand of respiration-related elements. Furthermore, null *HOG1* mutants in *C. albicans* have shown enhanced respiratory basal rate and increased mitochondrial ATP dependence compared to parental strains (Alonso-Monge et al., 2009).

Early *SEF1* induction in the *Dhhog1*Δ points to a transient or partial Hog1 involvement in the coordination of the iron-regulon and *RIB* gene expression, suggesting that riboflavin overproduction reflects internal metabolic imbalance rather than external iron limitation. Importantly, cells were not pre-cultured under iron-starvation conditions, and intracellular iron stores may have buffered iron uptake in both strains. Nevertheless, riboflavin was detected in the *Dhhog1*Δ supernatant as early as 27 h, suggesting that *Dh*Hog1 deletion may uncouple riboflavin accumulation from canonical iron-limitation signaling.

Sodium uptake remained comparable, possibly due to Hog1-independent sodium transporters (Almagro et al., 2001; Proft and Struhl, 2004; Velkova and Sychrova, 2006; Carcía-Salcedo et al., 2007; Montiel and Ramos, 2007). This indicates that riboflavin accumulation involves a broader reorganization of elemental homeostasis, likely related to increased energetic demands and precursor availability for riboflavin biosynthesis. From a metabolic standpoint, accelerated assimilation of phosphorus, sulfur, and magnesium may reflect increased biosynthetic and energetic demands that support riboflavin precursor availability. Phosphorus uptake is closely linked to ATP generation and nucleotide biosynthesis, and may contribute to sustaining the demand for purine-derived precursors such as GTP. Magnesium is broadly required as a cofactor for ATP-dependent enzymes and kinases, and is essential for multiple steps in central carbon metabolism and nucleotide metabolism, including pathways supplying ribulose-5-phosphate (Ru5P) and GTP. Since riboflavin biosynthesis depends on Ru5P and GTP as key precursors (Chatwell et al., 2006), and flux from the pentose phosphate pathway is expected to be a major determinant of riboflavin production capacity (Schlösser et al., 2001, 2007), enhanced macronutrient assimilation may be consistent with a physiological state favoring precursor generation in *Dhhog1*Δ. In addition, sulfur assimilation has been associated with riboflavin overproduction capacity in other riboflavinogenic yeasts; remarkably, deletion of *MET2* abolishes riboflavin overproduction in *Candida famata*, highlighting a functional link between sulfur/methionine metabolism and riboflavin biosynthesis regulation (Dmytruk et al., 2006). Moreover, *Dh*Hog1 absence could affect methionine and sulfur assimilation pathways, as observed in *C. albicans* and *S. cerevisiae* (Enjalbert et al., 2006; Separovich et al., 2024).

Notably, extracellular riboflavin accumulation was detected only when low pH and NaCl were combined, whereas neutralization of the starting pH abolished the phenotype even in the presence of NaCl, indicating a cooperative effect between acidity and salinity under the conditions tested. Concerning the role of pH on riboflavin secretion, reduced Vma1 activity (encoding the A subunit of the vacuolar ATPase) due to low extracellular pH (Diakov and Kane, 2010; Orij et al., 2011) could impair the vacuolar proton gradient and, consequently, diminish riboflavin influx into the vacuoles. The hypothesis that halted Vma1 activity contributes to riboflavin secretion could be further supported by the riboflavin excretion phenotype observed in *C. famata* null *VMA1* mutants (Andreieva et al., 2020a, b). Another possibility is that exposure to NaCl could enhance vacuolar fission in the *Dhhog1*Δ mutant, releasing riboflavin stored in vacuoles into the cytosol and facilitating secretion via plasma membrane riboflavin excretase Rfe1 (Tsyrulnyk et al., 2021). In *S. cerevisiae*, vacuolar fission has been reported in conditions of hyperosmotic, oxidative and endoplasmic reticulum stress (Gokbayrak et al., 2022). These stressors are expected to be exacerbated in a *hog1*Δ mutant, as suggested by previous reports in *D. hansenii*, *S. cerevisiae*, and *C. albicans* (Bicknell et al., 2010; Torres-Quiroz et al., 2010; Sánchez et al., 2020; Gokbayrak et al., 2022; Husain et al., 2022; de la Fuente-Colmenares et al., 2024). However, the role of vacuolar fission in riboflavin secretion remains unproven. Current evidence indicates that vacuolar storage of riboflavin has only been demonstrated in *A. gossypii*, where riboflavin protects spores against oxidative stress and UV radiation (Förster et al., 1999, 2001; Stahmann et al., 2001; Schlösser et al., 2007; Ledesma-Amaro et al., 2015; Silva et al., 2018). In this fungus, disruption of *VMA1* enhances riboflavin secretion (Förster et al., 1999, 2001). By contrast, while *RFE1* overexpression in *C. famata* increases riboflavin output, there is no evidence of vacuolar riboflavin accumulation in this species (Andreieva et al., 2020a, b). Importantly, Rfe1 is homologous to the mammalian riboflavin transporter *BCRP/ABCG2*, which exports riboflavin into extracellular fluids to prevent intracellular overaccumulation (Enjalbert et al., 2006; van Herwaarden et al., 2007; Pinto and Rivlin, 2013; Mao and Unadkat, 2015; Merrill and McCormick, 2020; Tsyrulnyk et al., 2020). Therefore, the contribution of vacuolar physiology and *RFE1* to riboflavin secretion in yeasts remains unresolved and requires further study.

In addition to the potential contribution of vacuolar physiology, combined low pH and salinity are also expected to reshape cellular energetics and ion-homeostasis, which can strongly influence metabolite transport and secretion. Beyond transcriptional responses, the combined low pH and salinity conditions are expected to impose strong energetic and ion-homeostasis constraints that may influence metabolite transport and secretion. Acidic pH can promote cytosolic proton accumulation, and this effect may be exacerbated in the presence of NaCl, thereby increasing the energetic demand required to maintain the plasma membrane potential and the cytosolic pH (Sychrová et al., 1999). In this context, Na^+^/H^+^ antiporters involved in Na^+^ efflux and intracellular sequestration may play key roles in adaptation (Velkova and Sychrova, 2006), and cation transport systems are known to be strongly induced under saline conditions (Almagro et al., 2001; Proft and Struhl, 2004; Carcía-Salcedo et al., 2007; Montiel and Ramos, 2007). Therefore, the enhanced extracellular riboflavin accumulation observed in *Dhhog1*Δ cells under low pH and NaCl may reflect altered energetic requirements and transporter activity under combined stress conditions, contributing to riboflavin export. Future studies directly assessing membrane potential/proton gradients and transporter contribution will be required to validate this hypothesis.

In parallel with these physiological constraints, our promoter and expression analyses suggest that stress-responsive transcription factors may also contribute to the observed transcriptional changes in key riboflavin biosynthesis-related genes. Promoter analysis suggests that *Dh*Hog1-regulated transcription factors, such as Hot1, Sko1, Skn7, Msn2/4, and Yap1 (Proft and Struhl, 2002; Ikner and Shiozaki, 2005; Auesukaree, 2017; Yaakoub et al., 2022), as well as the iron-responsive regulator Sef1, may contribute to the observed transcription responses; however, this analysis is predictive and does not provide experimental evidence of direct binding to *RIB* gene promoters. Under moderate saline stress (NaCl 0.6 M), *Dh*Hog1 is phosphorylated (active form) and normally channels metabolites toward glycerol (Sánchez et al., 2020). In its absence (*Dhhog1*Δ), glucose-6-phosphate availability may increase, which could enhance flux through the pentose phosphate pathway, promoting GTP and riboflavin biosynthesis. Activation of the glyoxylate cycle under NaCl stress, as previously reported in *D. hansenii*, may provide intermediates for glycine and riboflavin biosynthesis. In addition, the higher phosphate uptake observed in the *Dhhog1*Δ mutant suggests increased ATP demand and possible activation of the PHO pathway (Lau et al., 1998; Sánchez et al., 2008; Calahorra et al., 2009; Navarrete et al., 2021; Ruiz-Pérez et al., 2023), which would further support riboflavin production.

Together, these findings propose a hypothetical model in which *Dh*Hog1 coordinates environmental sensing (acidic pH and salinity) with nutrient assimilation and riboflavin metabolism. Loss of *Dh*Hog1 results in early riboflavin secretion, induction of *RIB1*, *RIB4*, *RIB6*, and *SEF1* genes, and accelerated uptake of phosphorus, sulfur, and magnesium, highlighting its role as a key component in the crosstalk between stress adaptation and secondary metabolism. A limitation of this study is that intracellular riboflavin levels were not quantified. Therefore, we cannot distinguish whether the *Dhhog1*Δ phenotype reflects increased intracellular biosynthesis, enhanced secretion/export, or both. Future studies quantifying intracellular flavin pools together with secretion rates will provide deeper mechanistic insight into *Dh*Hog1-dependent riboflavin dynamics under acidic and saline conditions. In addition, direct TF binding to *RIB/SEF1* promoters remains to be experimentally validated (e.g., ChIP-qPCR), to confirm the proposed *Dh*Hog1-dependent regulatory layer suggested by promoter and expression analyses.

In conclusion, *Dh*Hog1 modulates riboflavin biosynthesis in *D. hansenii*, preventing premature riboflavin accumulation during active growth. The lack of *Dh*Hog1 leads to early riboflavin secretion, upregulating *RIB1*, *RIB4*, *RIB6*, and *SEF1* genes, and accelerated assimilation of phosphorus, sulfur, and magnesium. This study highlights the importance of the HOG pathway as an integrator of environmental and metabolic signals in *D. hansenii*, extending its role beyond classical osmoregulation.

## Data Availability

The datasets presented in this study can be found in online repositories. The names of the repository/repositories and accession number(s) can be found in the article/Supplementary material.

## Footnotes

1
https://www.triton-lab.de/en/icp-oes

2
https://www.triton-lab.de/fileadmin/triton-lab/triton_lod.pdf

3
https://rsat.france-bioinformatique.fr/fungi/
