# Supplementary material for: Hog1 MAP kinase modulates early riboflavin accumulation under low-pH and saline conditions in Debaryomyces hansenii
Source: Front Microbiol. 2026 Feb 20;17:1746023. doi: 10.3389/fmicb.2026.1746023 (PMC12963334; doi:10.3389/fmicb.2026.1746023)
Supplement: Supplementary file 1 [file Data_Sheet_1.pdf]

>DhRIB1_upstream

aaatgtcgcacaattgctcaggaaccgtttaaacggcctaaatagaccactggatggtctgatatgatc
tactaagactcctccctcggtatcaaccgtagccacttgaaacaatgcccacgtacatacatgcctacg
ttggatagtttaatgtagtcaaatcaccggttcggacatgttgatttacacgtgaaatgaatggacctc
tagtccaccgtaactcacaattggctggattatttagacaatgaatacatgaaatgtatatcgctataa
gccttacgatctcgcctatggtttgacattgacattgcctctaccgttaaatttattgttcggtgccta
ttatctaatttctatcaaaagtgagctaaaaatccgaacccggtcaatcaaatagtaaatttcatcca
aattgtatggaacatttcttaattggatatatcaacagatatttaattagtatttctaacggcataaaa
caatttaaaagtgtcgaatatcctacttgattttaccataaatttgtaagtttgaataagacttatttc
aaggaattaagcaaattgtgtgatttgatactttcattagtaattgtgacaaattatagaacgcattca
aaat**ATG**

>DhRIB7_upstream

aacaaggcaagccatgaggtaaaggtagagaagactgacggcgatgagttcgatggcatagatgatgcc
gacgacatatatcccgaaaaggagtacaacgagtggagaatacgagagaaagacagatataaacgagat
agggaacgtataatgcaagaagaacagataaaggacgaaatagtgaggcgtggtaacttgacggaagaa
gaattgatatctaatttcaagaaaagacgtcaggaagaagattcagcgagttcttcgaagccgaagaat
taccataagggtgcgtttttcaatgatagcgaagacatcgacaagttattgaagagaacatacgaacaa
gttggagatgatgatgatgatgatgataatgctaaagatcattcaagacctacgaagttgaaatttaac
tagatatatatagagcacgattaatgacaatgataagggcctggctaacaggctatgacgacataaatc
ctaagacgtagacgtaggctggaatagtaagccaaatttcaagtactctaattcgcttgtacttcgacc
gcatcgaaaatcgatcggagatcaatttgaagtttatggcatattctcgaatttgataagacttctagc
atat**ATG**

>DhRIB2_upstream

ctacttctggcggcgaatccgattcgaatgaatccagtgataacgatgaatataacatgaaagatttgc
ctatcggcgttgatgggaaaccaattccctattggttatataaacttcaaggataccacaagacatacg
aatgcgaaatttgcggtaacacaacgtacaaaggtagggctgtgtttgctaagcatttcaatagtgcaa
agcaccaacacggtttaggttgcttaggtattagtgatgattacatggccttgtttaaaagcatagtta
aaattgatgaagcattagacttatggcgtagattaaaaaaggaaagaaggattaaagagggagatactg
aaaatgctattgaagttgaagatgaagaagggaatgtcttatccgaaaaggactacttggaattgaaga
aacaaggcctattgtaaagttgtacatagttagtcgaatatatgcgagtttagccgtctcggttatcgc
tgaaaaatttatgtaatccgatttttcatatttgcatttagatatattaaagcttcattacgctttgat
ggtaatatatagaaatagattattacactttaagtataatttctcccgctatttctataaaagttcaag
tcaa**ATG**

>DhRIB5_upstream

tacaacagaagctcttctcagtacgttagtaccatctaggcaccaaccaggcaccgcccagcaccacaa
acgtactacaaagacacttctatgtcatattaacctcctgatacaaacatactctgttgagcttaaagt
cggtgagcacagactttgcatgtcccgatgcaacacacctattcttgatctgactgtacgaagtgtatt
taggcatagctgttaactgtaactgtgcctccaatctaaccctagccagtaattcggtgtttctggcga
tatatcttaatgcatttctggtaacctcatgctcggcatactgtaatcttttgaattgatccttgatca
ctctaccattcacgtaacagtgtttaggaagttcgaattgaactgggaatctgaatggcattgtctatc
ctttacacagccttttcacaattttaaacggactagtctgtttgtactatacaatcggttcattggagg
acgatgaaaagtgaaaaattattggtcttaaatttacgcgtatcatagtacactacaaaacaccggtag
cgtaatcggtgattgcaaagtacataaggtgtcaaaatcatgactaattaccctcctatatagactgta
gatc**ATG**

>DhRIB6_upstream

aaatgtactcaaccgagcaaaatagagttatgacatagaatggttttcctatgtccgaaagtccacttc
attgaaataccgatcacaggcaaatcctcctactttcaaagggctttgattcctgtgaatcaacgattc
accgatattgacgatgctccccatcaaaactacctcccattggtttgactacgttcagtactacggtgt
atcgtccgacaatgtaccattctattgcccagttcttcgggttggtaacaaactataatatcacgtgaa
acttacaataacaatttcaataatagtccgttaaacggagaagatacaacgtataaaattagatacgaa
aattaggcttattgttgttatcatgtcaaattcaaaggctatgcctgtctaataatgcaaagtgaagtg
acagctcaattcattagccaaattcttctaattattagtaaaatttattccgactgcggatgaaaaat
aaatcagcagaaatatgaatcagcgagattcagaataacgaatttatataaggccctatatattgcat
ggtaacgatataaaatacagatattgtataaaccatatctctcagattaagactttcttaggtattagt
aaac**ATG**

>DhRIB4_upstream

ctctggccattgacggtcagccgctgaccttaacataatcgtctggtggatgccatcgctcaggaatgt
gtggaattaaccgtttctgcgcttgttagtactagttcgatctaaatcgacagcataattgtgaatcag
atagggggtgaaagactatttgatggtatttttatcttcatatatatcagttgaactatgcaattaatat
tttcatcatcatttcactgctgcattgtcggaatgttagaaatacatacttgaaaacaacttgagctaa
taaaaagatttcttccttctagacagaaatttttttgttcactatttatgttccagcagatgggcac
gagcttacaactggtagtgtgtttaccgtctgtcaccatcgccggaaatcgcactggctttgtgggctc
aattttaaccgcttattgtcggttaattatctctgattgtcgcaggctcactggggggttgtcactgggg
ggctttgaacatttgctgacattttgttcacgtgaccggacgaaacatcgggcataatgtgagcgtat
atatatgaacctggtatgggacaattttcagtccaaaaacgaatacatttagaaactacaacgaata
taag**ATG**

>DhSEF1_upstream

tgataattagccgtttatagtttgtaatctgcactaatacatatttgttcatttttacatcatttattg
ttctcttcgatgcattatcctcattcacataactatgatttcaattgccgtcaagctttaagccttgct
tattgtatgtttcgttagcctgtataataactcaccaaccgaaaggacgaacaaacgtaattactcaat
aaggaaatcaaaattatcttaacgatctgcgtactaaactatgccgtggaattgcgcaaatctgcgaaa
aatttttatcttttccatcaatcagtcgggtaactcccattgctgggcgtgtaacaccaattcccataa
aaatcatactggagacaaacactggcaactatagtacggaaaatttattcaatatagaatatatttgat
tatattagtctttgaataatatattgcccgtgttacatattcagcttatcctttcagatagtttgagtc
gtcgtttgaaatacaaaaatatactactgatttctaattgaaaatctcgaatcattcttaggtttgatc
tttttgataacaatattggaagcaagatttgaaagtttccgttaggaaagttagttaataagttatctt
tcaa**ATG**

Supplementary figure 2. Transcription factor putative binding sites in the upstream intergenic region 625 bp to the start codon. Transcription factor binding sites color code: Sef1, Sko1, Skn7 Yap1, Msn2/4, Hot1

>CaSef1_consensus_found_by_ChIP_by_Chen_etal_2011

TTCGGATCCGAAATCCG

>CfSef1_binding_site_in_CfRIB1_by_Romanov_etal_2025

TAAAAATCCGAACCCCGG

>RIB1_-606to-585

aggaaccgtttaaacggcctaa

>RIB1_-544to-523

tccctcggtatcaaccgtagcc

>RIB1_-468to-447

caaatcaccggttcggacatgt

>RIB1_-253to-232

aaaaatccgaaccccggtcaat

>RIB2_-621to-600

ttctggcggcgaatccgattcg

>RIB2_-165to-144

agtttagccgtctcggttatcg

>RIB5_-83to-62

acaccggtagcgtaatcggtga

>RIB6_-428to-407

actacggtgtatcgtccgacaa

>RIB6_-328to-307

aatagtccgttaaacggagaag

>RIB6_-168to-147

tttattccgactgcgggatgaa

>RIB4_-618to-597

cattgacggtcagccgctgacc

>RIB4_-256to-235

accgtctgtcaccatcgccgga

>RIB4_-207to-186

ttaaccgcttattgtcggttaa

>RIB4_-108to-87

gaccggacgaaacatcgggcat

Supplementary figure 3. Putative Sef1 binding sites in selected sequences containing putative Sef1 binding sites in *D. hansenii RIB* genes upstream intergenic regions. Ca = *Candida albicans*; Cf = *Candida famata.*

>CaSef1_consensus_found_by_ChIP_by_Chen_etal_2011

TTCGGATCCGAAATCCG------

>CfSef1_binding_site_in_CfRIB1_by_Romanov_etal_2025
TAAAAATCCGAACCCCGG-----

>RIB1_-468to-447
CAAAT-CACCGGTTCGGACATGT

>RIB2_-165to-144
AGTTT-AGCCGTCTCGGTTATCG

>RIB1_-253to-232
AAAAA-TCCGAACCCCGGTCAAT

>RIB6_-328to-307
AATAG-TCCGTTAAACGGAGAAG

>RIB6_-168to-147
TTTAT-TCCGACTGCGGGATGAA

>RIB4_-618to-597
CATTG-ACGGTCAGCCGCTGACC

>RIB1_-606to-585
AGGAAC-CGTTTAAACGGCCTAA

>RIB6_-428to-407
ACTACGGTGTATCGTCCGAC-AA

>RIB4_-207to-186
TTAACCGCTTATTGTCGGTT-AA

>RIB1_-544to-523
T-CCCTCGGTATCAACCGTAGCC

>RIB4_-256to-235

A-CCGTCTGTCACCATCGCCGGA

>RIB2_-621to-600

T-TCTGGCGGCGAATCCGATTCG

>RIB5_-83to-62

ACACCGGTAGCGTAATCGGTGA-

>RIB4_-108to-87

G-ACCGGACGAAACATCGGGCAT

Supplementary figure 4. Multiple alignment for Sef1 consensus binding motif logo generation. The alignments were made with Multalin version 5.4.1. Symbol comparison table: blosum62, with gap penalty at extremities. The logo was generated with WebLogo.
