# Supplementary material for: Hog1 MAP kinase modulates early riboflavin accumulation under low-pH and saline conditions in Debaryomyces hansenii
Source: Front Microbiol. 2026 Feb 20;17:1746023. doi: 10.3389/fmicb.2026.1746023 (PMC12963334; doi:10.3389/fmicb.2026.1746023)
Supplement: Supplementary file 2 [file Data_Sheet_2.pdf]

Supplementary Material S1. Multiple alignments of *D. hansenii CBS767, C. albicans SC5314* and *S. cerevisiae S288C* Rib and Sef1 amino acid sequences

Dh**Rib1,** ***Ca*****Rib1**
(DEHA2A12870p, AOW31038.1)

Identities:229/316(72%), Positives:269/316(85%), Gaps:7/316(2%)

Query 30   NFNNMPLLSPTLTPSQVPISETPQVPPKVSDEVRNALPIHGKLPEVRCMARARIPTTNGP 89
            + N +P+++P + + +P++ TPQ PP ++ +RN   +   LP V+C+ARARIPTT GP
Sbjct 32   DVNRIPMVTPKPSTNNLPVT-TPQPPPAITQSMRNKSGLPDSLPHVKCLARARIPTTQGP 90

Query 90   EIFLHLYENNIDNKEHLAIVFGEDIRSKTLFAKRPNDTQQDRMTRGAYVGKLFPGRVDAD 149
            +IFLHLYENNIDNKEHLAIVFGEDIRS++LF   P +TQQDRMTRGAY+G+L PGR AD
Sbjct 91   DIFLHLYENNIDNKEHLAIVFGEDIRSRSLFKHYPGETQQDRMTRGAYIGRLTPGRTIAD 150

Query 150 HDSHSNMSLNFDTDGDLIREPSTTFMEDPCIVRIHSECYTGETAWSARCDCGEQFDEAGR 209
             D +   L FD DG+LI + S T+ DP +VRIHSECYTGETAWSARCDCGEQFDEAGR
Sbjct 151 SDGTN--ELKFDKDGNLIID-SLTYT-DPTLVRIHSECYTGETAWSARCDCGEQFDEAGR 206

Query 210 IMGNDGHGCIVYLRQEGRGIGLGEKLKAYNLQDLGADTVQANLILRHPADGRSFSLATAI 269
            IMG GHGC+VYLRQEGRGIGLGEKLKAYNLQDLGADTV+ANLILRHPADGR+FSLATAI
Sbjct 207 IMGEAGHGCMVYLRQEGRGIGLGEKLKAYNLQDLGADTVEANLILRHPADGRNFSLATAI 266

Query 270 LLDLGLAEIKLLTNNPDKILAVEGKNRDIKVLERVPMVPLAWKTE--DGIKSKEIEGYLS 327
            L+DLGL EIKLLTNNPDKI+AVEGK++++KVLERVPMVPL+W + +GIKSKEIEGYLS
Sbjct 267 LVDLGLVEIKLLTNNPDKIVAVEGKHQEVKVLERVPMVPLSWGKDGKNGIKSKEIEGYLS 326

Query 328 TKIERMGHLLEKPLKI 343
            TKIERMGHLLEKP+KI
Sbjct 327 TKIERMGHLLEKPIKI 342

***Dh*Rib1, *Sc*Rib1**
(DEHA2A12870p, NP_009520.1)

Identities:189/288(66%), Positives:224/288(77%), Gaps:15/288(5%)

```
Query  70   GKLPEVRCMARARIPTTNGPEIFLHLYENNIDNKEHLAIVFGEDIRSKTLFAKRPNDTQQ  129
            G LP V+C+ARARIPTT GP+IFLHLY NN DNKEHLAIVFGEDIRS++LF +R  +TQQ
Sbjct  29   GGLPLVQCVARARIPTTQGPDIFLHLYSNNRDNKEHLAIVFGEDIRSRSLFRRRQCETQQ  88

Query  130  DRMTRGAYVGKLFPGRVDADHDSHSNMSLNF-DTDGDLIREPSTTF-MEDPCIVRIHSEC  187
            DRM RGAY+GKL+PGR  AD D    ++L F D+ G+L+   +TT+   +  +VRIHSEC
Sbjct  89   DRMIRGAYIGKLYPGRTVADEDDRLGLALEFDDSTGELLASKATTWDAHNDTLVRIHSEC  148

Query  188  YTGETAWSARCDCGEQFDEAGRIM-----------GNDGHGCIVYLRQEGRGIGLGEKLK  236
            YTGE AWSARCDCGEQFD AGR++           G +GHG IVYLRQEGRGIGLGEKLK
Sbjct  149  YTGENAWSARCDCGEQFDRAGRLIACDHEPTSNIKGGNGHGVIVYLRQEGRGIGLGEKLK  208

Query  237  AYNLQDLGADTVQANLILRHPADGRSFSLATAILLDLGLAEIKLLTNNPDKILAVEGKNR  296
            AYNLQDLGADTVQANL+L+HP D R FSL  AILLDLG+  ++LLTNNP+KI  V+
Sbjct  209  AYNLQDLGADTVQANLMLKHPVDARDFSLGKAILLDLGIGNVRLLTNNPEKIKQVDHAPY  268

Query  297  DIKVLERVPMVPLAW-KTEDGIKSKEIEGYLSTKIERMGHLLEKPLKI  343
             +K +ERVPMVP+ W  + +GI SKEIEGYL TKIERMGHLL +PLK+
Sbjct  269  -LKCVERVPMVPIHWTNSSEGIDSKEIEGYLRTKIERMGHLLTEPLKL  315
```

***Dh*Rib2, *Ca*Rib2**
(DEHA2E11374p, AOW29623.1)

Identities:375/584(64%), Positives:467/584(79%), Gaps:15/584(2%)

```
Query  1    MSKEEAKGVKRSPSPAEINPLLTPKLRDSLGFRMKRQTIDKRTLTAYQQLGGEESNDDTN  60
            +S   ++G+KR+ SP    P  + K+ D+ GFR+++Q IDK  +T+
Sbjct  18   LSLTLSRGMKRAASPRAEQPPHSKKVVDAKGFRVRQQNIDKHKITS-------------E  64

Query  61   TLSKTKTIHEEEAEGASYTIDGRLRRVNPYFFTYLTYCKMRWRDRKLLDIFVKEFRDRDA  120
            + +  K+IHEEE EGA+Y I+GRLRRV PYF+TYLTYCK+RW+DRKL+D+F+ EFRDR
Sbjct  65   STATQKSIHEEETEGANYVIEGRLRRVTPYFYTYLTYCKLRWQDRKLIDVFIDEFRDRTP  124

Query  121  DFYKKTIAAGQVTLNKKPADLDSIVRNGDLISHRCHRHEPSVSSRGIKIVHEDENIIAID  180
            D Y+K +  G V +N + A+L++I+RNGDLISHR +R EP VSSR IKIV ED++++ ID
Sbjct  125  DAYRKAVEEGLVKVNSQVANLETILRNGDLISHRSYRREPPVSSRDIKIVFEDDDLLVID  184

Query  181  KPSGIAVHPTGRYRYNTITKIFQHEFGKVVHPCNRLDRLTSGLMFLGKNAKGADAFVQQI  240
            KP GI VHPTGRYRYNT+TKIF+HE GK+VHPCNRLDRLTSGLMFLGK++KGA+  + QI
Sbjct  185  KPGGIPVHPTGRYRYNTVTKIFEHEKGKIVHPCNRLDRLTSGLMFLGKSSKGANNMMSQI  244

Query  241  RDRTVKKEYIARVVGKFDIGDVEVDKPLKTVSPKHGLNRVDFDEGKEAKTVFRRISYDPE  300
            RDR V KEYI+RV G+F +  + VDKPL T SPK  LN VD + GKEAKT F+R+SYDP
Sbjct  245  RDRNVTKEYISRVKGEFPLDRIIVDKPLSTKSPKLTLNVVDMENGKEAKTEFQRVSYDPI  304

Query  301  SDTSIVKCFPFTGRTHQIRVHLQYIGHAIANDPMYSNATVWGKNLGKHGEGENADIISKL  360
            ++TS+VKC P TGRTHQIRVHLQ++G+ IANDP+YS+  VWGKNLG++GE +   ++ +L
Sbjct  305  TNTSVVKCHPLTGRTHQIRVHLQFLGYPIANDPIYSSEFVWGKNLGENGEADLDQVMERL  364

Query  361  DRIGKDKASSTWIHPQEDGEILSGHLCDICSAELYTDPGPNDLDLWLHAYKYEAADKTWS  420
            D IGK +A+++WIHP+ DGE+    +C I    LY+D G NDLDLWLHAYKYEA DK+WS
Sbjct  365  DLIGKSRAATSWIHPEGDGEVALDEICPITGLPLYSDAGSNDLDLWLHAYKYEADDKSWS  424

Query  421  YKTEYPEWATSPHRKYMELALEMANKCGETQTQFNVGAVLVNNGEVLATGHSRELPGNTH  480
            YKTEYPEWA  P RK+M++A+E A KCGETQTQFNVG VLV+NG+V++TGHSRELPGNTH
Sbjct  425  YKTEYPEWALEPSRKFMKMAIEEAEKCGETQTQFNVGCVLVHNGQVISTGHSRELPGNTH  484

Query  481  AEQCALEKYFEQT-GKREVPAGTEIYTTMEPCSLRLSGNLPCVDRILET-NIKTCFVGVV  538
            AEQCALEKYF +  G+REVPAGTEI+T+MEPCSLRLSGNLPCVDRIL+T NIKTCFVGV+
Sbjct  485  AEQCALEKYFSKNGGEREVPAGTEIFTSMEPCSLRLSGNLPCVDRILQTKNIKTCFVGVL  544

Query  539  EPDIFVKNNSGYTKLTERDVEYIHIPGYEELCLKIAKKGHEKIE  582
            EPDIFVKNNS Y KL +  VEYIHIPGYEE CL+IAK+GHE I+
Sbjct  545  EPDIFVKNNSSYKKLLDHGVEYIHIPGYEETCLEIAKRGHEHIQ  588
```

***Dh*Rib2, *Sc*Rib2**
(DEHA2E11374p, NP_014575.1)

Identities:314/515(61%), Positives:380/515(73%), Gaps:16/515(3%)

```
Query  79   TIDGRLRRVNPYFFTYLTYCKMRWRDRKLLDIFVKEFRDRDADFYKKTIAAGQVTLNKKP  138
            TIDG LR++ PYFFTY T+CK RWRDRKL+D+FV EFRDR+  +Y KTIA G+V LN +P
Sbjct  73   TIDGPLRKIEPYFFTYKTFCKERWRDRKLVDVFVSEFRDREPSYYSKTIAEGKVYLNDEP  132

Query  139  ADLDSIVRNGDLISHRCHRHEPSVSSRGIKIVHEDENIIAIDKPSGIAVHPTGRYRYNTI  198
            A+LD+I+R+GDLI+H+ HRHEP V+S+ I IV EDE+I+ IDKPS I VHPTGRYR+NTI
Sbjct  133  ANLDTIIRDGDLITHKVHRHEPPVTSKPIDIVFEDEDILVIDKPSSIPVHPTGRYRFNTI  192

Query  199  TKIFQHEFGKVVHPCNRLDRLTSGLMFLGKNAKGADAFVQQIRDRTVKKEYIARVVGKFD  258
            TK+ + + G  VHPCNRLD+ TSGLMFL K   GAD    Q++ R V KEY+ARV G+F
Sbjct  193  TKMLERQLGYSVHPCNRLDKPTSGLMFLAKTPLGADRMGDQMKAREVTKEYVARVKGEFP  252

Query  259  IGDVEVDKPLKTVSPKHGLNRV---DFDEGKEAKTVFRRISYDPESDTSIVKCFPFTGRT  315
            IG VEVDKP+++V+PK  LN V     +  K AKTVF+R+SYD    TSIVKC P TGRT
Sbjct  253  IGIVEVDKPVRSVNPKVALNAVCEMSDENAKHAKTVFQRVSYD--GQTSIVKCKPLTGRT  310

Query  316  HQIRVHLQYIGHAIANDPMYSNATVWGKNLGKHGEGENADIISKLDRIGKDKASSTWIHP  375
            HQIRVHLQY+G  IANDP+YSN  +WG +LG+ G     DI+ KLD IGK   + +WIHP
Sbjct  311  HQIRVHLQYLGFPIANDPIYSNPDIWGPDLGRGGLQNYDDIVLKLDAIGKTNPAESWIHP  370

Query  376  QEDGEILSGHLCDICSAELYTDPGPNDLDLWLHAYKYEAADKT-------WSYKTEYPEW  428
              +GE L G  C+ C AE+YTDPG NDLDLWLHA++YE+ ++        WSY+T+YPEW
Sbjct  371  HSEGEYLLGRQCEECEAEMYTDPGTNDLDLWLHAFRYESLERNSDTQKPLWSYRTKYPEW  430

Query  429  ATSPHRKYMELALEMANKCGETQTQFNVGAVLVNNGEVLATGHSRELPGNTHAEQCALEK  488
            A  PHR+YME+A++ A KCG T+T F+VGAVLV+  +VLATG+SRELPGNTHAEQCAL K
Sbjct  431  ALEPHRRYMEMAVKEAGKCGPTKTAFSVGAVLVHGTQVLATGYSRELPGNTHAEQCALIK  490

Query  489  Y--FEQTGKREVPAGTEIYTTMEPCSLRLSGNLPCVDRILETN--IKTCFVGVVEPDIFV  544
            Y         VP GT +YTTMEPCS RLSGN PC DRIL T   I T FVGV+EPD FV
Sbjct  491  YSQLHPNCPTIVPMGTVLYTTMEPCSFRLSGNEPCCDRILATQGAIGTVFVGVMEPDTFV  550

Query  545  KNNSGYTKLTERDVEYIHIPGYEELCLKIAKKGHE  579
            KNN+   KL    V YI IPGYEE C  IA KGH+
Sbjct  551  KNNTSLNKLESHGVNYIQIPGYEEECTIIAFKGHD  585
```

***Dh*Rib4, *Ca*Rib4**
(DEHA2D04180p, AOW26224.1)

Identities:142/164(87%), Positives:156/164(95%), Gaps:0/164(0%)

```
Query  1    MAVKGLGKVDQQYDGSKLRIGILHARWNKKIIDSLVEGAVKKLQEFNVKPENIVVESVPG  60
            MAVKGLG+VDQ+YDGSKLRIGILHARWN+KIID+LV GAVK+LQEF VK ENI++E+VPG
Sbjct  43   MAVKGLGEVDQKYDGSKLRIGILHARWNRKIIDALVAGAVKRLQEFGVKEENIIIETVPG  102

Query  61   SFELPYGTKLFFDKQERLGEPLDAVIPIGVLIKGSTMHFEYICDSVTHQLMKLNFELNRP  120
            SFELPYG+KLF +KQ+RLG+PLDA+IPIGVLIKGSTMHFEYICDS THQLMKLNFEL  P
Sbjct  103  SFELPYGSKLFVEKQKRLGKPLDAIIPIGVLIKGSTMHFEYICDSTTHQLMKLNFELGIP  162

Query  121  VIFGVLTCLTDEQAEARAGLIEGKMHNHGEDWGAAAVEMCTKFD  164
            VIFGVLTCLTDEQAEARAGLIEGKMHNHGEDWGAAAVEM TKF+
Sbjct  163  VIFGVLTCLTDEQAEARAGLIEGKMHNHGEDWGAAAVEMATKFN  206
```

***Dh*Rib4, *Sc*Rib4**
(DEHA2D04180p, NP_014498.1)

Identities:111/164(68%), Positives:137/164(83%), Gaps:1/164(0%)

```
Query  1    MAVKGLGKVDQQYDGSKLRIGILHARWNKKIIDSLVEGAVKKLQEFNVKPENIVVESVPG  60
            MAVKGLGK DQ YDGSK+R+GI+HARWN+ IID+LV+GA++++    V+  NI++E+VPG
Sbjct  1    MAVKGLGKPDQVYDGSKIRVGIIHARWNRVIIDALVKGAIERMASLGVEENNIIIETVPG  60

Query  61   SFELPYGTKLFFDKQERLGEPLDAVIPIGVLIKGSTMHFEYICDSVTHQLMKLNFELNRP  120
            S+ELP+GTK F D+Q +LG+PLD VIPIGVLIKGSTMHFEYI DS TH LM L  +++ P
Sbjct  61   SYELPWGTKRFVDRQAKLGKPLDVVIPIGVLIKGSTMHFEYISDSTTHALMNLQEKVDMP  120

Query  121  VIFGVLTCLTDEQAEARAGLIEG-KMHNHGEDWGAAAVEMCTKF  163
            VIFG+LTC+T+EQA ARAG+ E   MHNHGEDWGAAAVEM  KF
Sbjct  121  VIFGLLTCMTEEQALARAGIDEAHSMHNHGEDWGAAAVEMAVKF  164
```

***Dh*Rib5, *Ca*Rib5**
(DEHA2D13926p, AOW29934.1)

Identities:159/237(67%), Positives:196/237(82%), Gaps:0/237(0%)

```
Query  1    MFTGLVEQVGTVLEYKDQDTSAAGGNGVSMTIGDCASILTDVHLGDSICTNGVCLTVTEF  60
            MFTGLVE +GTVL+Y   D S+ GG+GVS+TIG+C+ IL DV LGDSI TNGVCLTVTEF
Sbjct  1    MFTGLVETIGTVLDYSKHDDSSTGGDGVSITIGNCSEILEDVKLGDSISTNGVCLTVTEF  60

Query  61   NKEQTQFKVGIAPETLRRSNLGDLKVDSKVNLERAVTSDVRMGGHVVQGHVDTVAEITKR  120
            N  +T FKVG+APETLRR+NLGDLK  + VNLERAVTS+VR+GGH+VQGHVDT+A+I  +
Sbjct  61   NLGKTLFKVGVAPETLRRTNLGDLKHGAPVNLERAVTSEVRLGGHIVQGHVDTIAKIVDK  120

Query  121  VPDGNAITFTFRLRDKEYMSYIVEKGFIAIDGTSLTVTDVNYDTSEFSIMMVSYTQAKVI  180
             PDGNAI +TF LRDKE+++YIV KGFIAIDG SLTVT+V+    +FSIM++SY+Q KVI
Sbjct  121  KPDGNAIAYTFELRDKEFINYIVHKGFIAIDGASLTVTNVDPSKVQFSIMLISYSQEKVI  180

Query  181  MPMKHEGATVNIEVDLTGKLIEKQIEINLANQIENENSALSKLISSIIDRKLSQLSK  237
            +  K  G TVNIEVDLTGK IEKQ+EINL  QIEN +S L+KLISS++++K+ +  K
Sbjct  181  LAKKEVGDTVNIEVDLTGKFIEKQVEINLTGQIENSDSPLNKLISSLVEKKVKEYIK  237
```

***Dh*Rib5, *Sc*Rib5**
(DEHA2D13926p, NP_009815.1)

Identities:141/234(60%), Positives:173/234(73%), Gaps:4/234(1%)

```
Query  1    MFTGLVEQVGTVLEYKDQDTSAAGGNGVSMTIGDCASILTDVHLGDSICTNGVCLTVTEF  60
            MFTG+VE +GTVLE    D S +GG GVS+TIG+  SILTD H+GDSI  NGVCLTVTEF
Sbjct  1    MFTGIVECMGTVLENNPYDDSESGGQGVSITIGNAGSILTDCHVGDSIAVNGVCLTVTEF  60

Query  61   NKEQTQFKVGIAPETLRRSNLGDLKVDSKVNLERAVTSDVRMGGHVVQGHVDTVAEITKR  120
            N +   FKVGI+PET++RSN+      ++VNLERAV+ DVR GGH VQGHVDTVA I  R
Sbjct  61   NNDS--FKVGISPETIKRSNVASWIQGTQVNLERAVSQDVRFGGHYVQGHVDTVANIVSR  118

Query  121  VPDGNAITFTFRLRDKEYMSYIVEKGFIAIDGTSLTVTDVN--YDTSEFSIMMVSYTQAK  178
             P+GN+I F F+LRD+EY  YIVEKGFI IDGTSLT+  V+       F I M+ +TQ
Sbjct  119  RPEGNSIIFGFQLRDQEYFKYIVEKGFICIDGTSLTIIKVDPLSQGGAFYISMIKHTQDN  178

Query  179  VIMPMKHEGATVNIEVDLTGKLIEKQIEINLANQIENENSALSKLISSIIDRKL  232
            VIMP+K  G  VNIEVDLTGK+IEKQI + L NQI  ++S L+ +IS+II+ K+
Sbjct  179  VIMPLKKIGDEVNIEVDLTGKIIEKQILLTLENQISKKDSTLNTMISNIIEEKV  232
```

Dh**Rib6,** ***Ca*****Rib3**
(DEHA2G09504p, AOW26854.1)

Identities:161/204(79%), Positives:180/204(88%), Gaps:0/204(0%)

```
Query  1    MSAKFVSIPEAIEAFKNGEYLIVMDDESRENEGDLIMSAELMTQEKMAFLVRYSSGYVCV  60
            M+  F  I EA+EA+KNGE+LIVMDDE RENEGDLIM+AEL+TQEKMAFLVRYSSGYVCV
Sbjct  4    MTNIFTPIEEALEAYKNGEFLIVMDDEDRENEGDLIMAAELITQEKMAFLVRYSSGYVCV  63

Query  61   PLSTERADALNLHPMLANETDRHGTAYTVTCDYADGTTTGISAHDRALTSTKLADPNSKP  120
            PLS ERA+ L L PMLAN +DRHGTAYT+TCD+A+GTTTGISAHDRALT+  LA+PNSKP
Sbjct  64   PLSEERANQLELPPMLANRSDRHGTAYTITCDFAEGTTTGISAHDRALTTRSLANPNSKP  123

Query  121  VDFIRPGHILPLRAVPGLLKKRRGHTEAGVQLCELAGLQPAAVICEMVRDEDGLMMRLDD  180
             DFI+PGHILPLRAVPGLLKKRRGHTEA VQL  LAGLQPA VICE+VRDEDGLMMRLDD
Sbjct  124  QDFIKPGHILPLRAVPGLLKKRRGHTEAAVQLSTLAGLQPAGVICELVRDEDGLMMRLDD  183

Query  181  CTKFSKTHNIKMITIEQLVEHISQ  204
            C +F K H IK+I I QLVE+IS+
Sbjct  184  CIQFGKKHGIKIININQLVEYISK  207
```

***Dh*Rib6, *Sc*Rib3**
(DEHA2G09504p, NP_010775.1)

Identities:118/208(57%), Positives:152/208(73%), Gaps:9/208(4%)

```
Query  5    FVSIPEAIEAFKNGEYLIVMDDESRENEGDLIMSAELMTQEKMAFLVRYSSGYVCVPLST  64
            F  I +AIE FK  +++IVMDD  RENEGDLI +AE ++ E+MAFLVR+SSGYVC P++
Sbjct  2    FTPIDQAIEHFKQNKFVIVMDDAGRENEGDLICAAENVSTEQMAFLVRHSSGYVCAPMTN  61

Query  65   ERADALNLHPML-------ANETDRHGTAYTVTCDYADGTTTGISAHDRALTSTKLADPN  117
              AD L+L P+L        +N+ DRHGTAYT+T D A GTTTGISAHDR++T   LAD +
Sbjct  62   AIADKLDL-PLLRTGMKFESNDDDRHGTAYTITVDVAQGTTTGISAHDRSMTCRALADSS  120

Query  118  SKPVDFIRPGHILPLRAVPGLLKKRRGHTEAGVQLCELAGLQPAAVICEMVR-DEDGLMM  176
            S P  F++PGHI PLRA  G + +RRGHTEAGV LC+L+GL P AVI E+V  DE G MM
Sbjct  121  STPKSFLKPGHICPLRAADGGVLQRRGHTEAGVDLCKLSGLSPVAVIGELVNDDEQGTMM  180

Query  177  RLDDCTKFSKTHNIKMITIEQLVEHISQ  204
            RL+DC  F K H I +I+IE+L +++ +
Sbjct  181  RLNDCQAFGKKHGIPLISIEELAQYLKK  208
```

***Dh*Rib7, *Ca*Rib7**
(DEHA2G10010p, XP_711559.2)

Identities:156/300(52%), Positives:195/300(65%), Gaps:54/300(18%)

```
Query  1    MSLLPLTPSLRPFLEEYLPR---PCSN-----RPFVTLTYAQSLDSRIAAKPGEQTKISH  52
            MSL+PL  SL PFL+ YLP      +N     RPFVTLTYAQSLDS+IAA+PG QTK+SH
Sbjct  1    MSLIPLPESLIPFLDPYLPNIKVTTTNNGSLKRPFVTLTYAQSLDSKIAAQPGTQTKLSH  60

Query  53   LETKTMTHYIRSKHDGIMVGIGTVLADDPKLNCR--FEAEDGN---ISTP-RPIILDPTG  106
            LETKTMTHY+RSKHD I+VGIGT+LADDPKLNCR  FE+   N   IS   RP+++DP G
Sbjct  61   LETKTMTHYLRSKHDAILVGIGTILADDPKLNCRYIFESVKPNEQMISHQIRPVVIDPHG  120

Query  107  KWAYHKSQLRSVCDNN------------------------KGLAPFILIDETVTPRNEDV  142
            KW YH+SQL  +C  +                        GLAPFI+IDE+  P  E
Sbjct  121  KWQYHQSQLCQICQPSTTTTTTTTKKKKKNSESSPVSVPVPAGLAPFIIIDESTIPNIESE  180

Query  143  EVLDKQDGAFVRLPLLRNA---DKVG-NWNIILKKLFQLGIKSIMVEGGASIINDLLVYS  198
            +++ KQ G +++LPLL +     K+  NW  IL+KL+QLG+KSIM+EGGA IINDLL  +
Sbjct  181  KMVIKQGGKYIKLPLLLSTTTKTKISDNWREILQKLYQLGLKSIMIEGGAKIINDLLSIN  240

Query  199  ------------KIIDSLIITIGPVFLGKDGVEVSPSGHAGLIDVKWWQGIQDSVLCARL  246
                        K+IDS+IITI PVFLG +GV V P  +  L D+ WW GIQDS++ AR+
Sbjct  241  NSTDDDDDDDGQKLIDSVIITIAPVFLGCNGVTVHPYHNVLLKDINWWTGIQDSIIAARI  300
```

***Dh*Rib7, *Sc*Rib7**
(DEHA2G10010p, NP_009711.3)

Identities:119/255(47%), Positives:160/255(62%), Gaps:23/255(9%)

```
Query  1    MSLLPLTPSLRPFLEEYLPRPCSNR----PFVTLTYAQSLDSRIAAKPGEQTKISHLETK  56
            MSL PL   L  FL+ YLP          PFVTLTYAQSLD+R++  PG +T ISH ETK
Sbjct  1    MSLTPLCEDLPQFLQNYLPNAGQTENTIVPFVTLTYAQSLDARVSRGPGVRTTISHPETK  60

Query  57   TMTHYIRSKHDGIMVGIGTVLADDPKLNCRFEAEDGNISTPRPIILDPTGKWAYHKSQLR  116
            TMTHY+R  HDGI+VG GTVLAD+P LNC++   D   ++PRPII+D   KW +  S+++
Sbjct  61   TMTHYLRHHHDGILVGSGTVLADNPGLNCKW-GPDPAANSPRPIIIDTKQKWRFDGSKMQ  119

Query 117   SVCDNNKGLAPFILIDETVTPRNEDVEVLDKQDGAFVRLPLLRNADKVGNWNIILKKLFQ 176
             +    +G  P +++  T  P      ++ +Q   +   P + +  K+ +W    KKLF+
Sbjct  120  ELFIKRQGKPPIVVV--TSEP------IIKEQHVDYAICP-INDTTKLVDW----KKLFE  166

Query  177  L-----GIKSIMVEGGASIINDLLVYSKIIDSLIITIGPVFLGKDGVEVSPSGHAGLIDV  231
            +      I+S+MVEGGA++IN LL+ S I++SLIITIG  FLG  G EVSP     L D+
Sbjct  167  ILKEEFNIRSVMVEGGANVINQLLLRSDIVNSLIITIGSTFLGSSGTEVSPPQTVNLKDM  226

Query  232  KWWQGIQDSVLCARL  246
             WW+GI D VLCARL
Sbjct  227  SWWKGITDVVLCARL  241
```

Dh**Sef1, *****Ca*****Sef1**
(DEHA2C16676p, AOW30969.1)

Identities:594/980(61%), Positives:687/980(70%), Gaps:114/980(11%)

```
Query  1    MEKSRIKSILPKPSVSGGSP-------AGTTNGGGNKRRSL---PANNATVVKKQKSQSL  50
             EK +++ ILPKPS +  +P         T    +KR+S    P N +   +K  S +
Sbjct  3    FEKGKVR-ILPKPSPTPTNPQTPLPLLPAQTKPVNSKRKSAASTPGNESKKSRKSNSTAS  61

Query  51   SEAS----SMGQSGDKGSKQTGHRPVTSCTFCRQHKIKCNASDNYPNPCHRCDKMGLKCE  106
            +  S    S+G    K SK TGHRPVTSCTFCRQHKIKCNASDNYPNPC RC KMGLKCE
Sbjct  62   TPNSATPTSVGTPPQKTSKPTGHRPVTSCTFCRQHKIKCNASDNYPNPCERCKKMGLKCE  121

Query  107  IDPQFRPKKGSQIQSLKSDVDELRAKIEMLTKNESLLTQALNQHNMNFIQQQSQPTFSSM  166
            IDP+FRP+KGSQIQSLKSDVDEL+AKIEMLTKNESLLTQALNQHN+N   QQ Q + S
Sbjct  122  IDPEFRPRKGSQIQSLKSDVDELKAKIEMLTKNESLLTQALNQHNLNHASQQQQSSGSQS  181

Query  167  TQNQLYNTTPIQRSAGTVSDFSNGNTPSQFQSDNSPHSFASGFPHVNTSANLSPNNPLQG  226
             Q    N    QR+      +++ N+  Q    N     AS  P V
Sbjct  182  QQQHPPNP---QRALS----YTSANSSPQVAFSN-----ASPIPSVT-------------  216

Query  227  NQILNDAIPTSAPLGHILHETSTDNSPTMEKKYDSLGKLAREHEEEFETIPEFVLGDVSV  286
                  +I  +APL H     ++DNSP             E+ EE + I EF+LGDV++
Sbjct  217  ------SIQQNAPLTH----ENSDNSPYALN--------TPENIEELQPISEFILGDVTL  258

Query  287  PLDKANELHDRFITKHLPFLPIITSNSATELYHKSKLLFWSVILTAALSEPEPTLYMSLA  346
            PL++ANELHD+F+T HLPFLPII S SATELYHKS+LLFW+VILTA+LSEPEP LYMSLA
Sbjct  259  PLNRANELHDKFMTTHLPFLPIIISRSATELYHKSQLLFWAVILTASLSEPEPKLYMSLA  318

Query  347  SLIKHLAIETCWTHTPRSTHVIQALVVLSIWPLPNEKVLDDCSYRFIGLAKNLSLQLGLH  406
            SLIK LAIETCW  TPRSTHVIQAL++LSIWPLPNEKVLDDCSYRF+GLAKNLSLQLGLH
Sbjct  319  SLIKQLAIETCWIKTPRSTHVIQALIILSIWPLPNEKVLDDCSYRFVGLAKNLSLQLGLH  378

Query  407  RGGEFIQEFSRTQASLGPDAELWRTRTWLAVFFCEQFWSSVLGLPPSINTTDYLLENARV  466
            RGGEFIQEFSR Q SLGPDAE WRTR+WLAVFFCEQFWSS+LGLPPSINTTDYLLENARV
Sbjct  379  RGGEFIQEFSRNQVSLGPDAERWRTRSWLAVFFCEQFWSSLLGLPPSINTTDYLLENARV  438

Query  467  DQSLPKNFRCLISLSIFQCKLVNVMGISVTRPDGLLEPSNRAGSLNTLDRELERLKFKLP  526
            D+SLPKNFRCLISLSIFQCKLVN+MGISVTRPDGLLEPSNRAGSL+ LDRELERL+FKL
Sbjct  439  DKSLPKNFRCLISLSIFQCKLVNIMGISVTRPDGLLEPSNRAGSLSLLDRELERLRFKLQ  498

Query  527  IENGSSIEIYYLYIKLMICCFAFLPGTPIEDQVKYVSAAYHSATRIITVTSQMISSNTIS  586
             E G  IE+YYLYIKLMICCFAFLPGTPIEDQVKYVS AY SATRI+T+ S+M+  N IS
Sbjct  499  FEEGGPIEVYYLYIKLMICCFAFLPGTPIEDQVKYVSFAYLSATRIVTIVSKMV--NDIS  556

Query  587  LIEFPIYVRQAITYSVLMLFKLHLSRYLIDKYVDSSRQSIVTVHRLFRNTLSSWKELQND  646
            LIE PIY+RQA+TYSV MLFKLHLSRYLIDKYVDS+RQSIVTVHRLFRNTLSSWK+LQND
Sbjct  557  LIELPIYIRQAVTYSVFMLFKLHLSRYLIDKYVDSARQSIVTVHRLFRNTLSSWKDLQND  616

Query  647  ISRTAKVLENLNIVLYTYPEVFLSDNKEIGGSIITRMRSHLTASLFYDLVWCIHEARRRT  706
            ISRTAKVLENLN+VLY YPE+FL+D++    SIITRMRSHLTASLFYDLVWC+HEARRR+
Sbjct  617  ISRTAKVLENLNMVLYNYPEIFLNDSENEDSSIITRMRSHLTASLFYDLVWCVHEARRRS  676

Query  707  LTDKSHPPEDKRVKNEPREDNDVSSFSNQRKPLPLPFYNQITKDDFKTITTTTPNGTTVT  766
            + DK      + + +P          +K LPLPFYNQITKDDFKTITTT+PNGTT+T
Sbjct  677  VLDKG------KRQAQP-----------NKKILPLPFYNQITKDDFKTITTTSPNGTTIT  719

Query  767  TLVPTDHAMTQAKSNANAIGLDKPLEINGIPLAMLEATGSMRDIDSRNTNQDTTINSSSV  826
            TLVPTD AM QAKS +      KPLEINGIPL MLEATGS R++     +Q     ++
Sbjct  720  TLVPTDQAMNQAKSKS--FDSSKPLEINGIPLPMLEATGSTREVLDSLPSQSLPSQAPTL  777

Query  827  PISNYPINNSNMVSSVSKT-----------------------QYQPQPIEQMPSGQSMLFS  863
               +     S  +                       Q+Q Q +Q P  Q  + S
Sbjct  778  QQYPMQQDQQQQEPSQQQQQKHSQQSQQYQQQQQSNQQQPHLQHQRQFQQSPPPQFSMIS  837

Query  864  PSDSLAEVHPVGTPNPANFQYFGG-----------NQSVINGVADQMDNFFQKQSDGWIN  912
             +  L +   +  +P    Y             ++ +   A Q+ NFF +Q+ GW N
Sbjct  838  STPPLQQPPFILANSPLPQTYLPKIDEMNMSPEVKQENSVAPFASQITNFFDQQTSGWFN  897

Query  913  NDNYQDDDFLGWFDANMIPE  932
            NDN QDDDFLGWFD NM+ E
Sbjct  898  NDN-QDDDFLGWFDVNMMQE  916
```

***Dh*Sef1, *Sc*Sef1**
(DEHA2C16676p, NP_009487.2)

Range 1: 333 to 946
Identities:317/625(51%), Positives:423/625(67%), Gaps:61/625(9%)

```
Query  276  IPEFVLGDVSVPLDKANELHDRFITKHLPFLPIITSNSATELYHKSKLLFWSVILTAALS  335
            + EFVLGD+S+ ++KAN LH  F+T++LP+ PI+ SN+ATELY +S+LLFW+V+LTA LS
Sbjct  333  VDEFVLGDISISIEKANRLHHIFVTRYLPYFPIMYSNNATELYSQSQLLFWTVMLTACLS  392

Query  336  EPEPTLYMSLASLIKHLAIETCWTHTPRSTHVIQALVVLSIWPLPNEKVLDDCSYRFIGL  395
            +PEPT+Y  L+SLIK LAIETCW  TPRSTH+ QAL++L IWPLPN+KVLDDCSYRF+GL
Sbjct  393  DPEPTMYCKLSSLIKQLAIETCWIRTPRSTHISQALLILCIWPLPNQKVLDDCSYRFVGL  452

Query  396  AKNLSLQLGLHRGGEFIQEFSRTQASLGPDAELWRTRTWLAVFFCEQFWSSVLGLPPSIN  455
            AK+LS QLGLHRG EFI EF+RTQ S+ P+AE WRTRTWL +FF E  W+S+LGLPP+ +
Sbjct  453  AKSLSYQLGLHRG-EFISEFTRTQTSM-PNAEKWRTRTWLGIFFAELCWASILGLPPT-S  509

Query  456  TTDYLLENA--------------------------------RVDQSLPKNFRCLISLSIF  483
             TDYLLE A                                  LP +FR L+SL+ F
Sbjct  510  QTDYLLEKALSCGDEESEEDNNDSIDNNNNDKRNKKDEPHVESKYKLPGSFRRLLSLANF  569

Query  484  QCKLVNVMGISVTRPDGLLEPSNRAGSLNTLDRELERLKFKLPIENGSSIEIYYLYIKLM  543
            Q KL +++G S + PDGLLEP  RA +L+ L +EL+ L   L  ++  ++ IY+LY+KL
Sbjct  570  QAKLSHIIGSSTSSPDGLLEPKYRAETLSILGKELDLLAKTLNFQSDDTVNIYFLYVKLT  629

Query  544  ICCFAFLPGTPIEDQVKYVSAAYHSATRIITVTSQMISSNTISLIEFPIYVRQAITYSVL  603
            +CCFAFLP TP  DQ+ YV+ AY +AT+I+T+ + ++   T  LIE PIY+RQA T+S L
Sbjct  630  VCCFAFLPETPPTDQIPYVTEAYLTATKIVTLLNNLLE--THQLIELPIYIRQAATFSAL  687

Query  604  MLFKLHLSRYLIDKYVDSSRQSIVTVHRLFRNTLSSWK-ELQNDISRTAKVLENLNIVLY  662
            +LFKL L+  L DKY DS+RQS+VT+HRL+RN L++W   ++NDISRTA +LE LN VL
Sbjct  688  ILFKLQLTPLLPDKYFDSARQSVVTIHRLYRNQLTAWATSVENDISRTASMLEKLNFVLI  747

Query  663  TYPEVFLSDNKEIGGSIITRMRSHLTASLFYDLVWCIHEARRRTLT-DKSHPPEDKRVKN  721
             +PEVF+ ++      II+RMRSHLT SLFYDLVWC+HEARRR +  + +    +K  K
Sbjct  748  MHPEVFVEED-----GIISRMRSHLTGSLFYDLVWCVHEARRREMDPEYNKQALEKAAKK  802

Query  722  EPREDNDVSSFSN------QRKPLPLPFYNQITKDDFKTITTTTPNGTTVTTLVPTDHAM  775
                N + + ++       RK  PLP YN I++DDF+T+T TTP+GTTVTTLVPT +A+
Sbjct  803  RKFSSNGIYNGTSSTGGITDRKLYPLPLYNHISRDDFETVTKTTPSGTTVTTLVPTKNAL  862

Query  776  TQAKSNANAIGLDKP---LEINGIPLAMLEATGSMR-------DIDSRNTNQDTTINSSS  825
             QA+  A     D     +EINGIPL+ML  TGS++         +S + N + T+  +S
Sbjct  863  KQAEKLAKTNNGDSDGSIMEINGIPLSMLGETGSVKFQSLFANTSNSNDYNNNRTLLDAS  922

Query  826  VPISNYPINNSNMVSSVSKTQYQPQ  850
             IS  P N+   V+SV  +   PQ
Sbjct  923  NDIS-IPSNSIYPVASVPASNNNPQ  946
```

Range 2: 46 to 131
Identities:54/86(63%), Positives:69/86(80%), Gaps:0/86(0%)

```
Query  64   SKQTGHRPVTSCTFCRQHKIKCNASDNYPNPCHRCDKMGLKCEIDPQFRPKKGSQIQSLK  123
            S Q  HRPVTSCT CRQHKIKC+AS N+P+PC RC+K+GL CEI+PQFRPKKGSQ+Q L+
Sbjct  46   SHQINHRPVTSCTHCRQHKIKCDASQNFPHPCSRCEKIGLHCEINPQFRPKKGSQLQLLR  105

Query  124  SDVDELRAKIEMLTKNESLLTQALNQ  149
             DVDE+++K++ L  N+S+    L Q
Sbjct  106  QDVDEIKSKLDTLLANDSVFVHLLQQ  131
```

Range 3: 1097 to 1144
Identities:22/50(44%), Positives:32/50(64%), Gaps:2/50(4%)

```
Query  880   ANFQYFGGNQSVINGVADQMDNFFQKQSDGWINNDNYQDDDFLGWFDANM  929
             +NFQ    N++ +    +++ +FFQ+QS GWI   N  +DDF GWFD NM
Sbjct  1097  SNFQTID-NENNVKTPGNKLTDFFQQQSAGWIEG-NSSNDDFFGWFDMNM  1144
```
