# Supplementary material for: Hog1 MAP kinase modulates early riboflavin accumulation under low-pH and saline conditions in Debaryomyces hansenii
Source: Front Microbiol. 2026 Feb 20;17:1746023. doi: 10.3389/fmicb.2026.1746023 (PMC12963334; doi:10.3389/fmicb.2026.1746023)
Supplement: Supplementary file 3 [file Data_Sheet_3.pdf]

Supplementary table 1. Element concentration in the supernatants

Element	WT						*Dhhog1Δ*					
	Log phase			Stationary phase			Log phase			Stationary phase		
	ICP units		µM	ICP units		µM	ICP units		µM	ICP units		µM
Cl	15648	mg/l	4.41E+05	14499	mg/l	4.09E+05	15501	mg/l	4.37E+05	14280	mg/l	4.03E+05
Na	11390	mg/l	4.95E+05	10710	mg/l	4.66E+05	11320	mg/l	4.92E+05	10336	mg/l	4.50E+05
Ca	32	mg/l	7.98E+02	34	mg/l	8.48E+02	35	mg/l	8.73E+02	26	mg/l	6.49E+02
Mg	51	mg/l	2.10E+03	52	mg/l	2.14E+03	55	mg/l	2.26E+03	40	mg/l	1.65E+03
K	295	mg/l	7.55E+03	260	mg/l	6.65E+03	292	mg/l	7.47E+03	259	mg/l	6.62E+03
Br	2	mg/l	2.50E+01	2	mg/l	2.50E+01	3	mg/l	3.75E+01	1	mg/l	1.25E+01
B	1	mg/l	9.25E+01	1	mg/l	9.25E+01	1	mg/l	9.25E+01	1	mg/l	9.25E+01
F	0.01	mg/l	5.26E-01	0	mg/l	0.00E+00	0.01	mg/l	5.26E-01	0	mg/l	0.00E+00
Sr	0	mg/l	-	0	mg/l	-	0	mg/l	-	0	mg/l	-
S	1034	mg/l	3.22E+04	1068	mg/l	3.33E+04	1058	mg/l	3.30E+04	884	mg/l	2.76E+04
Li	0	µg/l	-	0	µg/l	-	0	µg/l	-	0	µg/l	-
Ni	0	µg/l	-	0	µg/l	-	0	µg/l	-	0	µg/l	-
Mo	35	µg/l	3.65E-01	26	µg/l	2.71E-01	18	µg/l	1.88E-01	21	µg/l	2.19E-01
V	0	µg/l	-	0	µg/l	-	0	µg/l	-	0	µg/l	-
Zn	89	µg/l	1.36E+00	47	µg/l	7.19E-01	67	µg/l	1.02E+00	50	µg/l	7.65E-01
Mn	108	µg/l	1.97E+00	102	µg/l	1.86E+00	112	µg/l	2.04E+00	80	µg/l	1.46E+00
I	222	µg/l	1.75E+00	222	µg/l	1.75E+00	222	µg/l	1.75E+00	222	µg/l	1.75E+00
Cr	0	µg/l	-	0	µg/l	-	0	µg/l	-	0	µg/l	-
Co	0	µg/l	-	0	µg/l	-	0	µg/l	-	0	µg/l	-
Fe	8	µg/l	1.43E-01	12	µg/l	2.15E-01	15	µg/l	2.69E-01	13	µg/l	2.33E-01
Ba	10	µg/l	7.28E-02	10	µg/l	7.28E-02	10	µg/l	7.28E-02	7	µg/l	5.10E-02
Be	0	µg/l	-	0	µg/l	-	0	µg/l	-	0	µg/l	-
Si	100	µg/l	3.56E+00	106	µg/l	3.77E+00	154	µg/l	5.48E+00	116	µg/l	4.13E+00
P	61902800	µg/l	2.00E+06	54056400	µg/l	1.75E+06	66704700	µg/l	2.15E+06	33927200	µg/l	1.10E+06
PO_4	189422.56	mg/l	1.99E+06	165412.58	mg/l	1.74E+06	204116.38	mg/l	2.15E+06	103817.23	mg/l	1.09E+06
Cu	7	µg/l	1.10E-01	0	µg/l	0.00E+00	7	µg/l	1.10E-01	0	µg/l	0.00E+00
